# Longitudinal Prediction of Infant MR Images With Multi-Contrast Perceptual Adversarial Learning

**DOI:** 10.3389/fnins.2021.653213

**Published:** 2021-09-09

**Authors:** Liying Peng, Lanfen Lin, Yusen Lin, Yen-wei Chen, Zhanhao Mo, Roza M. Vlasova, Sun Hyung Kim, Alan C. Evans, Stephen R. Dager, Annette M. Estes, Robert C. McKinstry, Kelly N. Botteron, Guido Gerig, Robert T. Schultz, Heather C. Hazlett, Joseph Piven, Catherine A. Burrows, Rebecca L. Grzadzinski, Jessica B. Girault, Mark D. Shen, Martin A. Styner

**Affiliations:** ^1^Department of Computer Science, Zhejiang University, Hangzhou, China; ^2^Department of Psychiatry, UNC School of Medicine, University of North Carolina, Chapel Hill, NC, United States; ^3^Department of Electrical and Computer Engineering Department, University of Maryland, College Park, MD, United States; ^4^Department of Information Science and Engineering, Ritsumeikan University, Shiga, Japan; ^5^Department of Radiology, China-Japan Union Hospital of Jilin University, Changchun, Jilin, China; ^6^Montreal Neurological Institute, McGill University, Montreal, QC, Canada; ^7^Department of Radiology, University of Washington, Seattle, WA, United States; ^8^Department of Speech and Hearing Sciences, University of Washington, Seattle, WA, United States; ^9^Mallinckrodt Institute of Radiology, Washington University School of Medicine, St Louis, MO, United States; ^10^Department of Psychiatry, Washington University School of Medicine, St. Louis, MO, United States; ^11^Department of Computer Science and Engineering, New York University, New York, NY, United States; ^12^Center for Autism Research, Department of Pediatrics, Children's Hospital of Philadelphia, and University of Pennsylvania, Philadelphia, PA, United States; ^13^Carolina Institute for Developmental Disabilities, University of North Carolina School of Medicine, University of North Carolina-Chapel Hill, Chapel Hill, NC, United States; ^14^Department of Pediatrics, University of Minnesota, Minneapolis, MN, United States; ^15^UNC Neuroscience Center, University of North Carolina-Chapel Hill, Chapel Hill, NC, United States; ^16^Department of Computer Science, University of North Carolina, Chapel Hill, NC, United States

**Keywords:** generative adversarial networks, MRI, longitudinal prediction, machine learning, infant, postnatal brain development, autism, imputation

## Abstract

The infant brain undergoes a remarkable period of neural development that is crucial for the development of cognitive and behavioral capacities (Hasegawa et al., 2018). Longitudinal magnetic resonance imaging (MRI) is able to characterize the developmental trajectories and is critical in neuroimaging studies of early brain development. However, missing data at different time points is an unavoidable occurrence in longitudinal studies owing to participant attrition and scan failure. Compared to dropping incomplete data, data imputation is considered a better solution to address such missing data in order to preserve all available samples. In this paper, we adapt generative adversarial networks (GAN) to a new application: longitudinal image prediction of structural MRI in the first year of life. In contrast to existing medical image-to-image translation applications of GANs, where inputs and outputs share a very close anatomical structure, our task is more challenging as brain size, shape and tissue contrast vary significantly between the input data and the predicted data. Several improvements over existing GAN approaches are proposed to address these challenges in our task. To enhance the realism, crispness, and accuracy of the predicted images, we incorporate both a traditional voxel-wise reconstruction loss as well as a perceptual loss term into the adversarial learning scheme. As the differing contrast changes in T1w and T2w MR images in the first year of life, we incorporate multi-contrast images leading to our proposed 3D multi-contrast perceptual adversarial network (MPGAN). Extensive evaluations are performed to assess the qualityand fidelity of the predicted images, including qualitative and quantitative assessments of the image appearance, as well as quantitative assessment on two segmentation tasks. Our experimental results show that our MPGAN is an effective solution for longitudinal MR image data imputation in the infant brain. We further apply our predicted/imputed images to two practical tasks, a regression task and a classification task, in order to highlight the enhanced task-related performance following image imputation. The results show that the model performance in both tasks is improved by including the additional imputed data, demonstrating the usability of the predicted images generated from our approach.

## 1. Introduction

The early postnatal period (neonate to one year of age) is a period of dynamic and rapid brain development with dramatic appearance changes in magnetic resonance images (MRI). This period has been associated with early atypical developmental trajectories in neurodevelopmental disorders, such as autism spectrum disorder (ASD) and schizophrenia (Hazlett et al., 2017; Gilmore et al., 2018). Longitudinal MRI allows the quantification of developmental trajectories over time and plays a critical role in neuroimaging studies of early brain development (Gilmore et al., 2012). However, missing data points is a common issue in longitudinal studies due to MRI scan failure, scheduling issues, or general participant attrition (Laird, 1988). Discarding those study participants with incomplete data significantly reduces the sample size and may even lead to unacceptable levels of bias (Matta et al., 2018). One solution to deal with the issue of missing data is to interpolate/extrapolate the missing data, called data imputation, from the data that is available. Such data imputation can be performed either at the image or at the measurement level.

Low rank matrix completion is a commonly proposed approach for measurement level imputation, for example, Thung et al. (2016) employed it to impute the missing volumetric features. A series of machine learning based approaches also have been proposed in this field, such as Meng et al. (2017) proposed the Dynamically-Assembled Regression Forests (DARF) in order to predict cortical thickness maps at missing time points. Rekik et al. (2016) developed a 4D varifold-based learning framework to predict the cortical shape at the time point in the first year of life using the cortical surface shape at birth. Additionally, a lot of variants of geodesic models (Fishbaugh et al., 2013, 2014; Fletcher, 2013; Singh et al., 2013a) were proposed for longitudinal shape imputation and regression. Compared with measurement-level methods, image-level methods directly predict the image appearance at a missing time point. In (Niethammer et al., 2011; Singh et al., 2013b), the geodesic models were used for longitudinal regression of related image appearance. And Rekik et al. (2015) proposed a sparse patch-based metamorphosis learning framework for regression of MRI appearance and anatomical structures with promising yet limited results.

In this paper, we focus on image-level approaches for infant longitudinal MRI prediction and we treat it as an image synthesis problem, i.e., synthesizing/predicting a missing MR image from an existing image of the same subject at a later or earlier time point. Recently, generative adversarial networks (GANs) have shown great potential in generating visually-realistic images for both natural image synthesis, e.g., in image-to-image translation (Isola et al., 2017; Liu et al., 2017; Yi et al., 2017; Zhu et al., 2017; Huang et al., 2018; Xiong et al., 2019; Emami et al., 2021), generating new plausible samples (Goodfellow et al., 2014; Zhang et al., 2019), generating photographs of human faces (Karras et al., 2017), and medical image synthesis, e.g., cross-modality synthesis (MR-to-CT Nie et al., 2017; Wolterink et al., 2017; Jin et al., 2019, MR-to-PET Pan et al., 2018, 2019, PET-to-MR Choi and Lee, 2018, CT-to-PET Ben-Cohen et al., 2017; Bi et al., 2017; Armanious et al., 2020, 3T-to-7T Qu et al., 2019), cross-site synthesis (Zhao et al., 2019), and multi-contrast MRI synthesis (Dar et al., 2019; Yang et al., 2020). Recently, GANs have also been applied to longitudinal MR image prediction. For example, Xia et al. (2019) proposed a conditional GAN that conditioned on age and health state (status of Alzheimer's Disease) to predict brain aging trajectories. In (Bowles et al., 2018; Ravi et al., 2019), a GAN is used to predict the Alzheimer's related brain degeneration from existing MR images, where biological constraints associated with disease progression are integrated into the framework. These longitudinal prediction approaches are limited to 2D T1w MRI that are hard to generalize to 3D, as well as having been designed for adult brain images related to Alzheimer's disease. Besides, we also notice that GAN-architectures with perceptual loss have been used in a few medical image applications. For example, Armanious et al. applied GAN with perceptual loss to PET-CT translation, MR motion correction and the PET denoising (Armanious et al., 2020). Dar et al. used a GAN with VGGNet-based perceptual loss for a multi-contrast MRI synthesis task (mapping among T1w MRI and T2w MRI) (Dar et al., 2019). Due to the nature of their tasks, they only focus on single modality 2D data. Also, they utilized VGGNet for the perceptual loss computation. Since VGGNet is a pretrained model based on 2D natural images, it may be not that appropriate for medical image tasks.

In this work, we propose a novel GAN adaptation for a new application: the longitudinal prediction of infant MR images in the first year of life. Since human brain size and shape changes rapidly in the first year of life, 2D methods are not suitable in our task. Thus, we present a fully 3D-based approach for the prediction of infant MR images. In addition, because of the myelination process, the infant brain shows a dramatic change of tissue contrast and anatomical structural shape, which further poses difficulties for prediction. While generative adversarial networks can produce images with realistic textures by enforcing the outputs from the generator to be close to the real data distribution, it cannot ensure the consistency between the outputs and the desired ground-truth images, so that the appearance of predicted image may look different from the ground-truth image. To handle the large variation in appearance, we add a voxel-wise reconstruction constraint, i.e., an L1 loss, to explicitly guide the generator to produce images that match ground-truth images at the voxel level. Although global structures can be well-preserved by harnessing L1 loss, it often results in an over-smoothed output (Pathak et al., 2016). Hence, to alleviate this issue, we also enhance our GAN with a perceptual loss term to maintain appearance consistency at the feature level. We propose to utilize Model Genesis (Zhou et al., 2019), which is a pre-trained model for 3D medical images, for this feature extraction. Finally, in order to tackle the reduced tissue contrast during the first year of life, particularly at about 6 months of age, we propose a multi-contrast framework, so that the complementary information of different contrasts (T1w and T2w images) can be exploited. The source code of our method will be released to the public upon acceptance of this manuscript at https://github.com/liying-peng/MPGAN.

Our main contributions are summarized as follows:

- To the best of our knowledge, this is the first application of deep generative methods for longitudinal prediction of structural MRI in the first year of life.
- Unlike previous 2D-based methods, our method is based on a 3D MRI prediction, where the volumetric spatial information is fully considered.
- To predict sharp, realistic and accurate images, we adopt a GAN with adversarial, pixel-wise reconstruction and perceptual loss. The perceptual loss is computed via features extracted from an application-specific model that has been pre-trained on 3D medical images.
- To leverage complementary information from multi-contrast data, we propose a novel multi-contrast framework to jointly predict T1w and T2w images.
- Extensive experiments demonstrate the effectiveness of our approach for use in longitudinal MRI prediction and imputation in the developing infant brain. We show that when using these imputed MR images to expand the training data in two practical machine learning tasks, we improve the model performance.

The remainder of this paper is organized as follows. In section 2, we introduce the experimental datasets and describe the methodological details of our proposed approach. Experimental results are presented in section 3 and then discussed in section 4. The conclusions are shown in section 5.

## 2. Methods

In this section, we first introduce a brief background of generative adversarial networks. Subsequently, we formulate our problem and then define our objective functions. Finally, our network architectures are discussed.

### 2.1. Generative Adversarial Network

The Generative Adversarial Network (GAN) is a generative deep learning model that was proposed by Goodfellow et al. (2014). The aim of it on image-to-image translation tasks is to learn a mapping from the input image *x* to the target image *y*, i.e., *x* → *y*. It consists of two separate components, each a neural network, specifically a generator *G* and a discriminator *D* network. In the training stage, these two networks compete with each other, where a) *G* attempts to fool *D* by generating a fake image *G*(*x*) that looks similar to a real target image *y*, and b) *D* aims to distinguish between the real image *y* and the fake image *G*(*x*). As the two networks face off, *G*(*x*) generates more realistic images that get closer to the real data distribution and *D*(*x*) becomes more skilled at differentiating images. At the end, the algorithm will converge to a Nash equilibrium (Nash et al., 1950). This two-player *minmax* game is formulated as: $\min_{G}\max_{D}L_{adv}\left(G,D\right)$, where the adversarial loss $L_{adv}$ can be defined as.

(1) $$\begin{array}{l} L_{adv}\left(G,D\right)={𝔼}_{x}\left[{\left(1-D\left(G\left(x\right)\right)\right)}^{2}\right]+{𝔼}_{y}\left[D{\left(y\right)}^{2}\right] \end{array}$$

### 2.2. Objective Design

We consider two settings in this work: (1) a single-input-single-output setting when using single contrast images and (2) a multi-input-multi-output setting when using multiple contrasts jointly. In the former setting, suppose ${\left\{x_{i},y_{i}\right\}}_{i=1}^{N}$ is a series of paired instances, where *x*_*i*_ is a T1w or a T2w image at age *a*_1_, *y*_*i*_ is the corresponding T1w or T2w image at age *a*_2_ and *N* is the number of paired subjects in the training set. Our goal is to learn the mapping *G*:*x* → *y*. In the latter setting, assume ${\left\{x_{i}^{T1},x_{i}^{T2},y_{i}^{T1},y_{i}^{T2}\right\}}_{i=1}^{N}$ is a set of paired subjects, where $x_{i}^{T1},x_{i}^{T2}$ indicate the T1w and T2w images at age *a*_1_ and $y_{i}^{T1},y_{i}^{T2}$ stand for the corresponding T1w and T2w images at age *a*_2_. The aim is then to learn two mapping functions: $G_{T1}:\left\{x^{T1},x^{T2}\right\}\to y^{T1}$ and $G_{T2}:\left\{x^{T1},x^{T2}\right\}\to y^{T2}$.

#### 2.2.1. Adversarial Loss

In the single-input-single-output setting, in order to learn the mapping *G*:*x* → *y*, we can employ the adversarial loss function of the original GAN (see Equation 1). In the multi-input-multi-output setting, the basic idea is same as the original GAN, but here we define two generators, i.e., $G_{T1}:\left\{x^{T1},x^{T2}\right\}\to y^{T1}$ and $G_{T2}:\left\{x^{T1},x^{T2}\right\}\to y^{T2}$. *G*_*T*1_ and *G*_*T*2_ aim at generating fake T1w and T2w images that look similar as real images, respectively. We also define two discriminators *D*_*T*1_ and *D*_*T*2_, where the intention of *D*_*T*1_ is to differentiate the real T1w image *y*^*T*1^ from the generated T1w image $G_{T1}\left(x^{T1},x^{T2}\right)$. Similarly, *D*_*T*2_ attempts to distinguish between *y*^*T*2^ and $G_{T2}\left(x^{T1},x^{T2}\right)$. With respect to generator *G*_*T*1_ and its discriminator *D*_*T*1_, the adversarial loss can be formulated as

(2) $$\begin{array}{l} L_{adv}\left(G_{T1},D_{T1}\right)={𝔼}_{x^{T1},x^{T2}}\left[{\left(1-D_{T1}\left(G_{T1}\left(x^{T1},x^{T2}\right)\right)\right)}^{2}\right]\\ \;+{𝔼}_{y^{T1}}\left[D_{T1}{\left(y^{T1}\right)}^{2}\right] \end{array}$$

The adversarial loss $L_{adv}\left(G_{T2},D_{T2}\right)$ can be expressed similarly.

#### 2.2.2. Voxel-Wise Reconstruction Loss

While the adversarial loss can optimize the generated output images closer to the real data distribution, it cannot ensure consistency between the outputs and the desired ground-truth images, so that a predicted image may not share the details of its corresponding ground-truth image. To deal with this problem, we further restrict the generator with a voxel-wise reconstruction loss. Here we choose a traditional L1 loss, as recommended in Zhao et al. (2015), which directly penalizes the voxel-wise differences between the two images. For the single-input-single-output setting, the voxel-wise reconstruction loss is given by

(3) $$\begin{array}{l} L_{vr}\left(G\right)={𝔼}_{x,y}\left[{\left\|y-G\left(x\right)\right\|}_{1}\right] \end{array}$$

For the multi-input-multi-output setting, the voxel-wise reconstruction loss is expressed as.

(4) $$\begin{array}{l} L_{vr}\left(G_{T1},G_{T2}\right)={𝔼}_{x^{T1},x^{T2},y^{T1}}\left[{\left\|y^{T1}-G_{T1}\left(x^{T1},x^{T2}\right)\right\|}_{1}\right]\\ \;+{𝔼}_{x^{T1},x^{T2},y^{T2}}\left[{\left\|y^{T2}-G_{T2}\left(x^{T1},x^{T2}\right)\right\|}_{1}\right] \end{array}$$

#### 2.2.3. Perceptual Loss

Although the voxel-wise reconstruction loss enforces voxelwise consistency between the real and generated images, it prefers an over-smoothed solution (Pathak et al., 2016). In other words, this loss commonly leads to outputs with well-preserved low-frequency information, e.g., global structures, at the expense of the high-frequency crispness. To alleviate this problem, we add a perceptual loss (Johnson et al., 2016) to the generator, which results in sharper images. The perceptual loss calculates the difference between two images in feature space in place of voxel space. Thus, it forces the generated images to be perceptually similar to the real images, instead of matching intensities exactly at the voxel level. Suppose ϕ_*m*_(*x*) is the output from the *m*-th layer of a feature extractor ϕ when processing the image *x*. For the single-input-single-output setting, the perceptual loss can be written as

(5) $$\begin{array}{l} L_{p}\left(G\right)={𝔼}_{x,y}\left[{\left\|\phi_{m}\left(y\right)-\phi_{m}\left(G\left(x\right)\right)\right\|}_{1}\right] \end{array}$$

For the multi-input-multi-output setting, the perceptual loss is formulated as

(6) $$\begin{array}{l} L_{p}\left(G_{T1},G_{T2}\right)={𝔼}_{x^{T1},x^{T2},y^{T1}}\left[{\left\|\phi_{m}\left(y^{T1}\right)-\phi_{m}\left(G_{T1}\left(x^{T1},x^{T2}\right)\right)\right\|}_{1}\right]\\ \;+{𝔼}_{x^{T1},x^{T2},y^{T2}}\left[{\left\|\phi_{m}\left(y^{T2}\right)-\phi_{m}\left(G_{T2}\left(x^{T1},x^{T2}\right)\right)\right\|}_{1}\right] \end{array}$$

**Overall objective:** By combining the above loss functions, we can define the final objective in the single-input-single-output setting as

(7) $$\begin{array}{l} \underset{G}{\min}\underset{D}{\max}L\left(G,D\right)=L_{adv}\left(G,D\right)+\alpha L_{vr}\left(G\right)+\beta L_{p}\left(G\right) \end{array}$$

Similar, we can define the total objective in the multi-input-multi-output setting as

(8) $$\begin{array}{l} \begin{array}{c} \underset{G_{T1},G_{T2}}{\min}\underset{D_{T1},D_{T2}}{\max}L\left(G_{T1},G_{T2},D_{T1},D_{T2}\right)=L_{adv}\left(G_{T1},D_{T1}\right)\\ +L_{adv}\left(G_{T2},D_{T2}\right)+\alpha L_{vr}\left(G_{T1},G_{T2}\right)+\beta L_{p}\left(G_{T1},G_{T2}\right) \end{array} \end{array}$$

where α and β are the coefficients to weight the loss contributions.

### 2.3. Network Architectures

#### 2.3.1. Perceptual Adversarial Network

**Figure 1:**
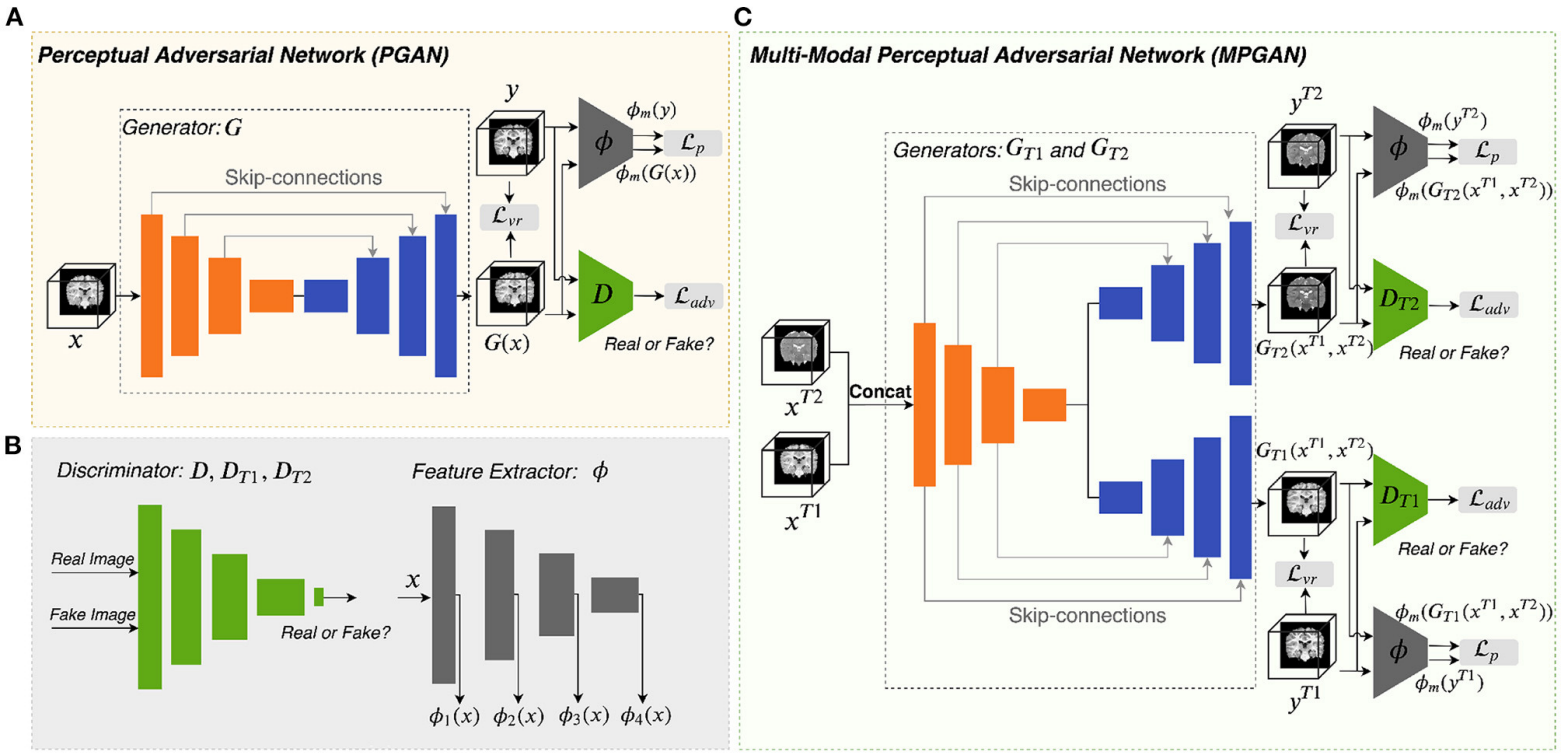
Overview of the proposed methods. Panels **(A,C)** are the architectures of PGAN and MPGAN, respectively. Panel **(B)** shows the discriminator and the feature extractor setup for the perceptual loss computation.

Figure 1A illustrates the architecture of the perceptual adversarial network (PGAN) that is designed for the single-input-single-output setting. It consists of a generator *G*, a discriminator *D* and a feature extractor ϕ. We utilize a traditional 3D-Unet (Çiçek et al., 2016) as generator. The 3D-Unet is an end-to-end convolutional neural network that was originally developed for medical image segmentation. It includes an analysis path (encoder) and a synthesis path (decoder). The encoder part contains four convolutional layers each of which includes two repeated 3 × 3 × 3 convolution operations, followed by a 2 × 2 × 2 max pooling for downsampling (except for the last layer). The decoder part is basically the same as the encoder part, but it replaces all downsampling with upsampling. Skip-connections are build between the layers of the encoder and their counterparts of the decoder. The architecture of our discriminator *D* is the same as the one in (Isola et al., 2017). It contains four stride-2 convolutional layers, with 64, 128, 256, and 512 channels, respectively. The output layer of it is a stride-1 convolutional layer with one channel, followed by a *sigmoid* activation function. Instance normalization (Ulyanov et al., 2016) is applied to the convolutional layers in both generator and discriminator.

For natural images, pretrained VGG networks are often adopted as the feature extractors. However, for medical image tasks, feature extractors based on pretrained VGG-Nets have the following limitations: (1) 3D medical images would have to be reformulated into a 2D format to fit VGG-Nets (2D networks), leading to the loss of rich 3D anatomical information. (2) Perceptual differences between natural projection images (such as photos) and 3D tomographic, medical images, are not captured. To overcome these limitations, we employ an existing application-specific model as our feature extractor ϕ, specifically Model Genesis (Zhou et al., 2019), which is built directly from 3D medical images. Note that Model Genesis is a U-Net style network and here we only use its encoder part for feature extraction. The details of PGAN are shown in Table 1.

**Table 1:** The network architecture of perceptual adversarial network.

**Generator**	
**Encoder network**	**Decoder (generator) network**
(3 × 3 × 3) 20 Conv, IN, RL	(2 × 2 × 2)↑, (3 × 3 × 3) 160 Conv, IN, RL
(3 × 3 × 3) 40 Conv, IN, RL, (2 × 2 × 2)↓	(3 × 3 × 3) 160 Conv, IN, RL
(3 × 3 × 3) 40 Conv, IN, RL	(2 × 2 × 2)↑, (3 × 3 × 3) 80 Conv, IN, RL
(3 × 3 × 3) 80 Conv, IN, RL, (2 × 2 × 2)↓	(3 × 3 × 3) 80 Conv, IN, RL
(3 × 3 × 3) 80 Conv, IN, RL	(2 × 2 × 2)↑, (3 × 3 × 3) 40 Conv, IN, RL
(3 × 3 × 3) 160 Conv, IN, RL, (2 × 2 × 2)↓	(3 × 3 × 3) 40 Conv, IN, RL
(3 × 3 × 3) 160 Conv, IN, RL	(1 × 1 × 1) 1 Conv, tanh
(3 × 3 × 3) 320 Conv, IN, RL	
**Discriminator**	
(4 × 4 × 4) 64 stride 2 Conv, LR, (4 × 4 × 4) 128 stride 2 Conv, IN, LR	
(4 × 4 × 4) 256 stride 2 Conv, IN, LR, (4 × 4 × 4) 512 stride 2 Conv, IN, LR	
(4 × 4 × 4) 1 stride 1 Conv, sigmoid	
**Feature extractor**	
(3 × 3 × 3) 32 Conv, IN, RL, (3 × 3 × 3) 64 Conv, IN, RL, (2 × 2 × 2)↓	
(3 × 3 × 3) 64 Conv, IN, RL, (3 × 3 × 3) 128 Conv, IN, RL, (2 × 2 × 2)↓	
(3 × 3 × 3) 128 Conv, IN, RL, (3 × 3 × 3) 256 Conv, IN, RL, (2 × 2 × 2)↓	
(3 × 3 × 3) 256 Conv, IN, RL, (3 × 3 × 3) 512 Conv, IN, RL	

*Conv, convolution; IN, Instance Normlization; RL, ReLu; LR, Leaky ReLU; ↓ and ↑, represent down- and upsampling, respectively; sigmoid, sigmoid activation function; tanh, tanh activation function*.

#### 2.3.2. Multi-Contrast Perceptual Adversarial Network

As shown in Figure 1C, the multi-contrast perceptual adversarial network (MPGAN) contains two generators *G*_*T*1_ and *G*_*T*2_, two discriminators *D*_*T*1_ and *D*_*T*2_ and one feature extractor ϕ. The feature extractor ϕ and the architectures of *D*_*T*1_ and *D*_*T*2_ are the same as for PGAN. *G*_*T*1_ and *G*_*T*2_ are both based on 3D-Unets that utilize a shared encoder and two independent decoders with skip-connections. The shared encoder learns complementary information from both T1w and T2w images and skip connections are used to transfer this information from the shared encoder to different decoders. We combine T1w and T2w images before feeding them into generators by applying a channel-wise concatenation.

## 3. Experiments and Results

### 3.1. Materials

The data used in this work is collected from the “Infant Brain Imaging Study” (IBIS) database (https://www.ibis-network.org) and the raw MR images are available on NDA (https://nda.nih.gov). All MR images were clinically evaluated by an expert neuroradiologist (RCM) and subjects with visible clinical pathology were excluded from the study. Data collection sites had approved study protocols by their Institutional Review Boards (IRB), and all enrolled subjects had informed consent provided by their parent/guardian. MR imaging parameters are as follows: (1) 3T Siemens Tim Trio at 4 sites; (2) T1w MRI: TR/TE = 2,400/3.16 ms, 256 × 256 × 160, 1 mm^3^ resolution; (3) T2w MRI: TR/TE = 3,200/499 ms, same matrix and resolution as T1w. A series of preprocessing steps were adopted, i.e., ICBM alignment, bias correction, geometry correction, skull stripping (see Hazlett et al., 2017 for details), and intensity normalization to range (−1,1). For our main dataset which is used for longitudinal prediction, a total of 289 subjects with two complete scans at 6 and 12 months were selected. The dataset was split into three sets: training set (231 subjects), validation set (29 subjects), and test set (29 subjects).

We also build two additional datasets to evaluate the applicability of our predicted/imputed images. In the first application, we aim at classifying subject image data into different Autism Diagnostic Observation Schedule social affect (ADOS-SA-CSS) based groups. Thus, only those subjects with valid ADOS-SA-CSS measures were employed. In addition, we reduced the size of the typical developing group for group size balancing. This resulted in 77 subjects with complete scans at 6 and 12 months and 103 subjects with scans either at 6 or 12 months. In the second application, we estimated a subject's gestational age (GA) at birth from its MRI data. Only subjects with known GA were selected and this resulted in 134 subjects with complete scan pairs at 6 and 12 months, as well as 76 subjects with scans either at 6 or 12 months. In both applications, we employ the imputed datasets as additional training data. No imputed images are used in the testing datasets.

### 3.2. Implementation Details

Our experiments were performed on a lambdalab GPU server with four NVIDIA TITAN RTX GPU with 24GB oncard memory. All the networks were implemented in Tensorflow and trained via Adam optimization (Kingma and Ba, 2014). The batch size was set to 1. The learning rate was initially set to 2e-4 for the first 44 epochs and decayed every 22 epochs with a base of 0.5 for an additional 176 epochs. The trade-off parameters α and β in Equation (7) and (8) were set to 25 and ϕ_1_(*x*) was used for computation of the perceptual loss based on a grid search. The details of the grid search are shown in the Supplementary Materials. Two longitudinal prediction tasks were performed in this work, i.e., prediction of 6-month images from 12-month images and prediction of 12-month images from 6-month images.

### 3.3. Alternative Networks for Comparison

In this paper, we also trained five additional networks for the purpose of comparison: (1) CycleGAN: 3D extension of original CycleGAN (Zhu et al., 2017). (2) Unet($L_{vr}$): 3D-Unet (Çiçek et al., 2016) trained with $L_{vr}$. (3) Unet ($L_{vr}+L_{p}$): 3D-Unet trained with both $L_{vr}$ and $L_{p}$. (4) GAN: original GAN (Goodfellow et al., 2014). (5) GAN+$L_{vr}$: original GAN with additional $L_{vr}$ term. To enable fair comparisons, we implemented these networks with parameters optimized the same way as our proposed methods. Further, the 3D-Unet was used as the backbone of Unet variant methods, i.e., (2) and (3), and it was also used as the generator of cycleGAN, GANs and our methods. The discriminators for (1), (4), and (5) are the same as for our models.

### 3.4. Evaluation via Appearance Based Metrics

In this section, images predicted by different methods are evaluated both in qualitative and quantitative fashion, focusing on the image appearance. In addition, we conducted a human perceptual study, where the participants were required to rate the predicted images based on visual realism and closeness to the ground truth images.

#### 3.4.1. Qualitative Results

**Figure 2:**
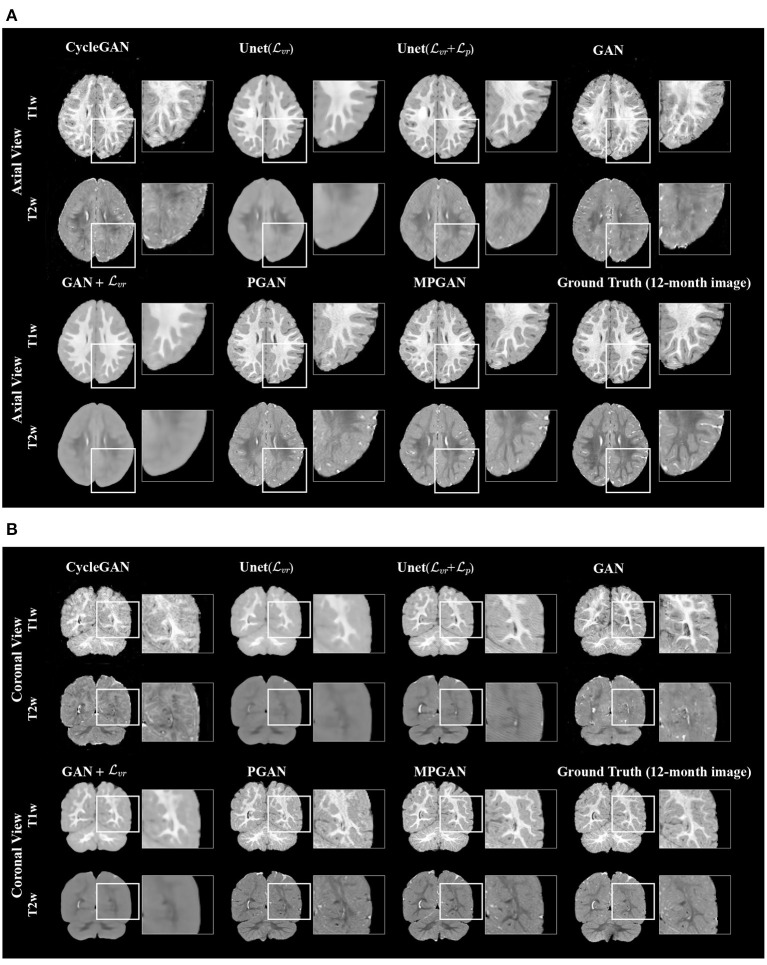
Examples of predicted MR images at 12 months (from 6 months MRI) compared across seven methods and the corresponding ground truth. **(A)** Axial view. **(B)** Coronal view.

**Figure 3:**
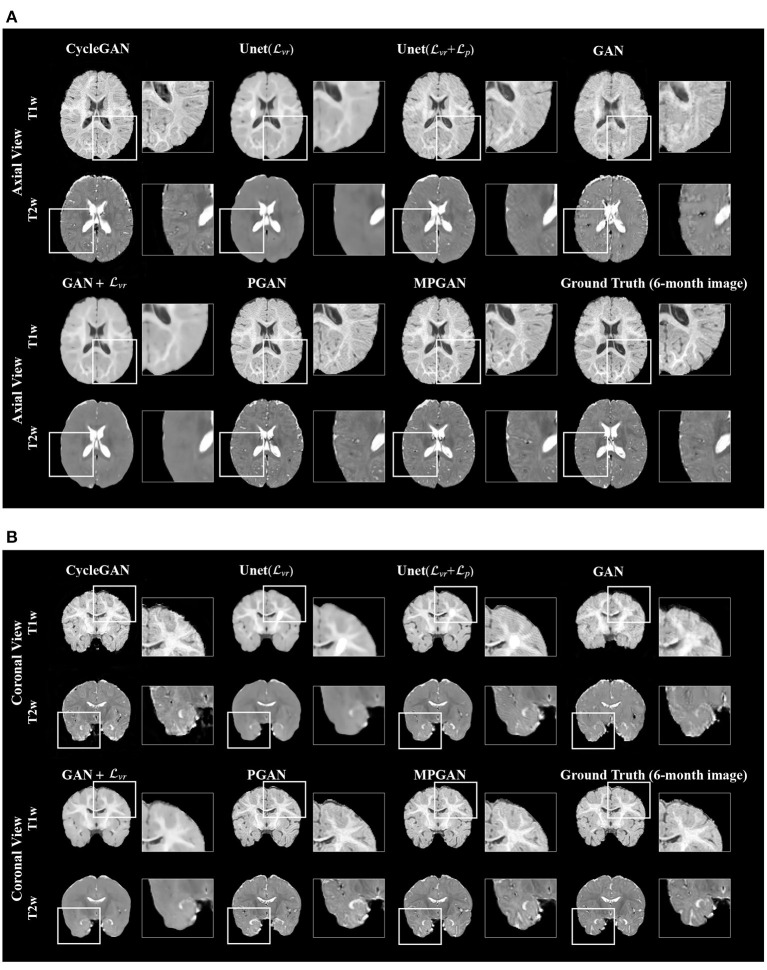
Examples of predicted MR images at 6 months (from 12 months MRI) compared across seven methods and the corresponding ground truth. **(A)** Axial view. **(B)** Coronal view.

The qualitative results for different methods are given in Figure 2 (6-to-12 months prediction task) and Figure 3 (12-to-6 months prediction task). The following findings were obtained from both tasks. (1) The images predicted by Unet($L_{vr}$) and GAN+$L_{vr}$ are globally consistent with the ground-truth images, but they appear overly smoothed, resulting in a poor visual quality. (2) Unet($L_{vr}$ + $L_{p}$) outperforms Unet($L_{vr}$) with the resultant images showing more high-frequency details. This indicates that adding the perceptual loss $L_{p}$ into training process helps the model to produce sharper details. However, the visual quality of the images generated by Unet($L_{vr}$ + $L_{p}$) is still unsatisfactory due to unrealistic textured appearance. (3) GAN produces the least anatomically accurate images, albeit with sharp details. This may be due to the reason that GAN is trained without any additional constraints to enforce appearance consistency between ground-truth and generated images. (4) Our PGAN and MPGAN show a superior performance compared with the other methods. They produce more realistic images with sharp and refined details from a visual perspective. (5) Compared to PGAN, MPGAN can predict finer details, especially for T2w images. This implies that multi-contrast learning can further improve the image quality by combining complementary information from T1w and T2w images.

#### 3.4.2. Quantitative Results

**Figure 4:**
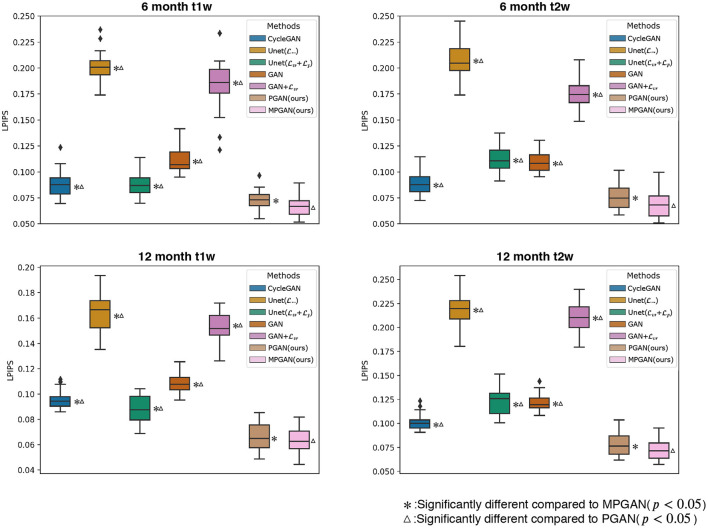
Quantitative comparison results of different methods based on Learned Perceptual Image Patch Similarity (LPIPS). A lower LPIPS value reflects a higher similarity between ground-truth and predicted images.

The development of optimal evaluation metrics for generated images is an challenging problem. Recently, a new learning-based metric, i.e., Learned Perceptual Image Patch Similarity (LPIPS), was proposed (Zhang et al., 2018) to assess the similarity between two images, which shows a superior performance compared to the traditional metrics. In this section, all of the methods are quantitatively compared based on LPIPS, which is shown in Figure 4. Note that LPIPS is a “similarity distance” calculated between the ground-truth image and the predicted image and lower value reflects a higher similarity. One can see that our PGAN and MPGAN give a notable improvement of LPIPS compared to other approaches, for both 6-to-12 months and 12-to-6 months prediction tasks. Specifically, MPGAN achieves the best performance. Paired *t*-tests showed statistically significant improvements (*p* < 0.05) of MPGAN over all other methods.

#### 3.4.3. Human Perceptual Study

We performed a perceptual study based on 116 sets of images, including 29 sets of 6-month T1w images, 29 sets of 6-month T2w images, 29 sets of 12-month T1w images, and 29 sets of 12-month T2w images. For each image set, the ground-truth image and the predicted images of seven different methods were shown to human raters for visual assessment. We asked 22 human raters (6 radiologists, 5 neuroscientists, 3 biomedical researchers, and 8 computer scientists with medical imaging background) to rate the image quality of the predicted images using a 7-point score, with 7 being the most realistic and closest to the ground-truth image (ties are allowed). All the images were shown initially in a random order and presented in axial, coronal, and sagittal views. The visualization order was continuously updated by sorting according to the current scores. The results of the perceptual study are shown in Table 2. Of all the studied methods, our MPGAN achieves the highest quality score across different images, with a statistical significance in Wilcoxon signed-rank test (*p* < 0.05 vs. other methods). The second-best performance is yielded by PGAN (*p* < 0.05 vs. other methods). While MPGAN are PGAN are close for T1w image prediction, MPGAN outperforms PGAN by a large margin for predicting T2w images (both 6 and 12 months), demonstrating the benefits of the multi-contrast architecture.

**Table 2:** The average score results of human assessments based on the appearance of images predicted by different methods.

**Method**	**Unet($L_{vr}$)**	**GAN+$L_{vr}$**	**GAN**	**Unet($L_{vr}+L_{p}$)**	**CycleGAN**	**PGAN**	**MPGAN**
6-month T1w	1.48 ± 0.64	2.18 ± 0.68	2.58 ± 1.09	3.44 ± 1.11	5.24 ± 0.91	6.13 ± 0.84	**6.43** **±** **0.72**
6-month T2w	1.87 ± 0.80	2.14 ± 0.78	3.29 ± 1.54	3.32 ± 0.84	5.34 ± 0.82	5.96 ± 0.76	**6.59** **±** **0.67**
12-month T1w	2.83 ± 1.27	2.15 ± 1.46	3.68 ± 1.75	4.20 ± 1.36	3.92 ± 1.64	6.22 ± 0.71	**6.64** **±** **0.64**
12-month T2w	2.13 ± 1.07	1.73 ± 0.88	2.63 ± 1.29	3.29 ± 1.06	4.01 ± 1.40	5.89 ± 0.64	**6.92** **±** **0.27**

*The scores range from 1 to 7, with higher scores indicating a higher degree of realism and closer appearance to the ground truth. Methods with best performance are bolded for each setting (significantly better than other measurements, p < 0.5)*.

### 3.5. Evaluation on Segmentation Task

**Figure 5:**
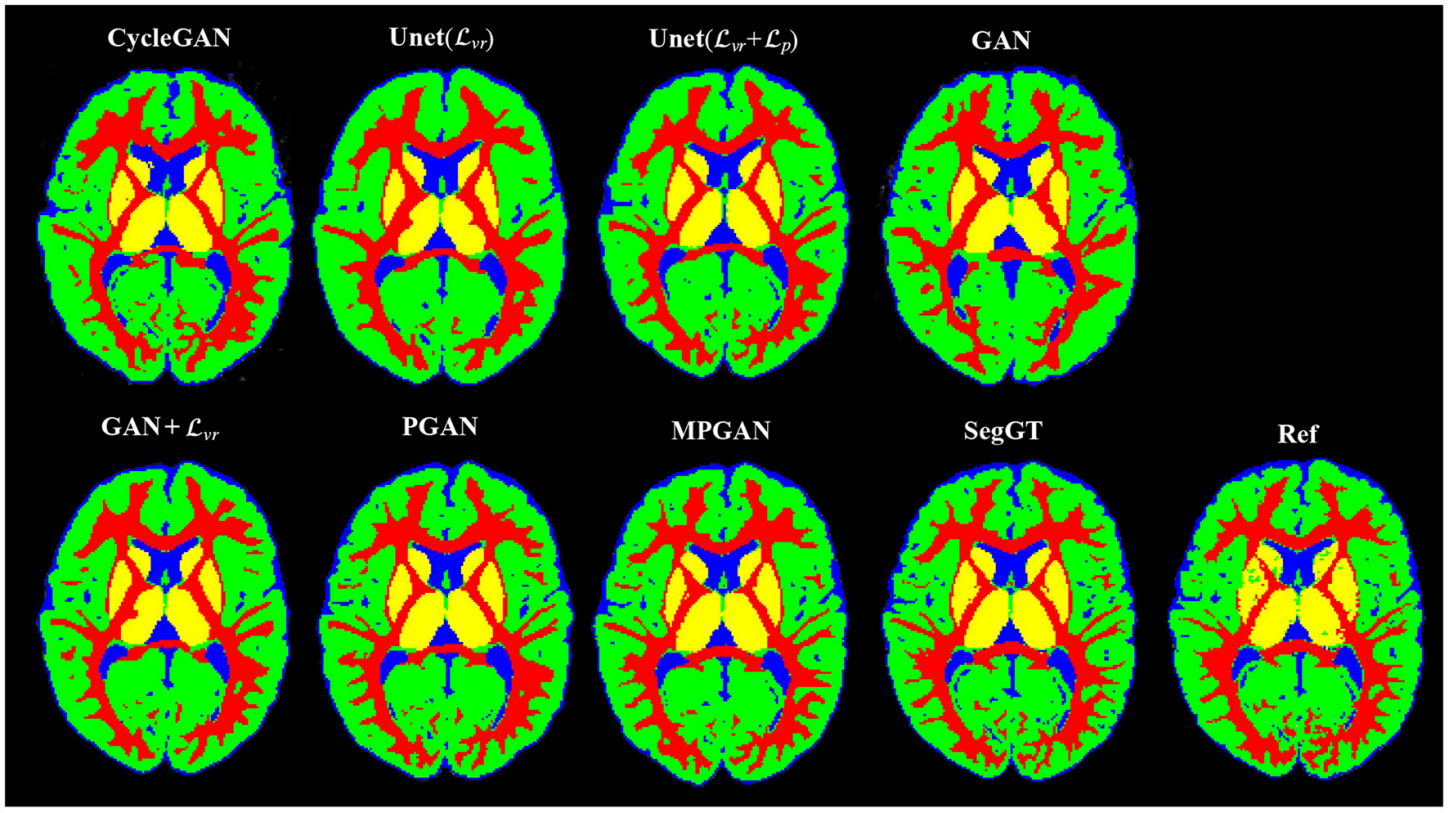
Examples of the tissue segmentation results. The first four columns are the automatic segmentations of the predicted and the ground-truth images (SegGT). The last column is the reference manual segmentation (Ref).

**Figure 6:**
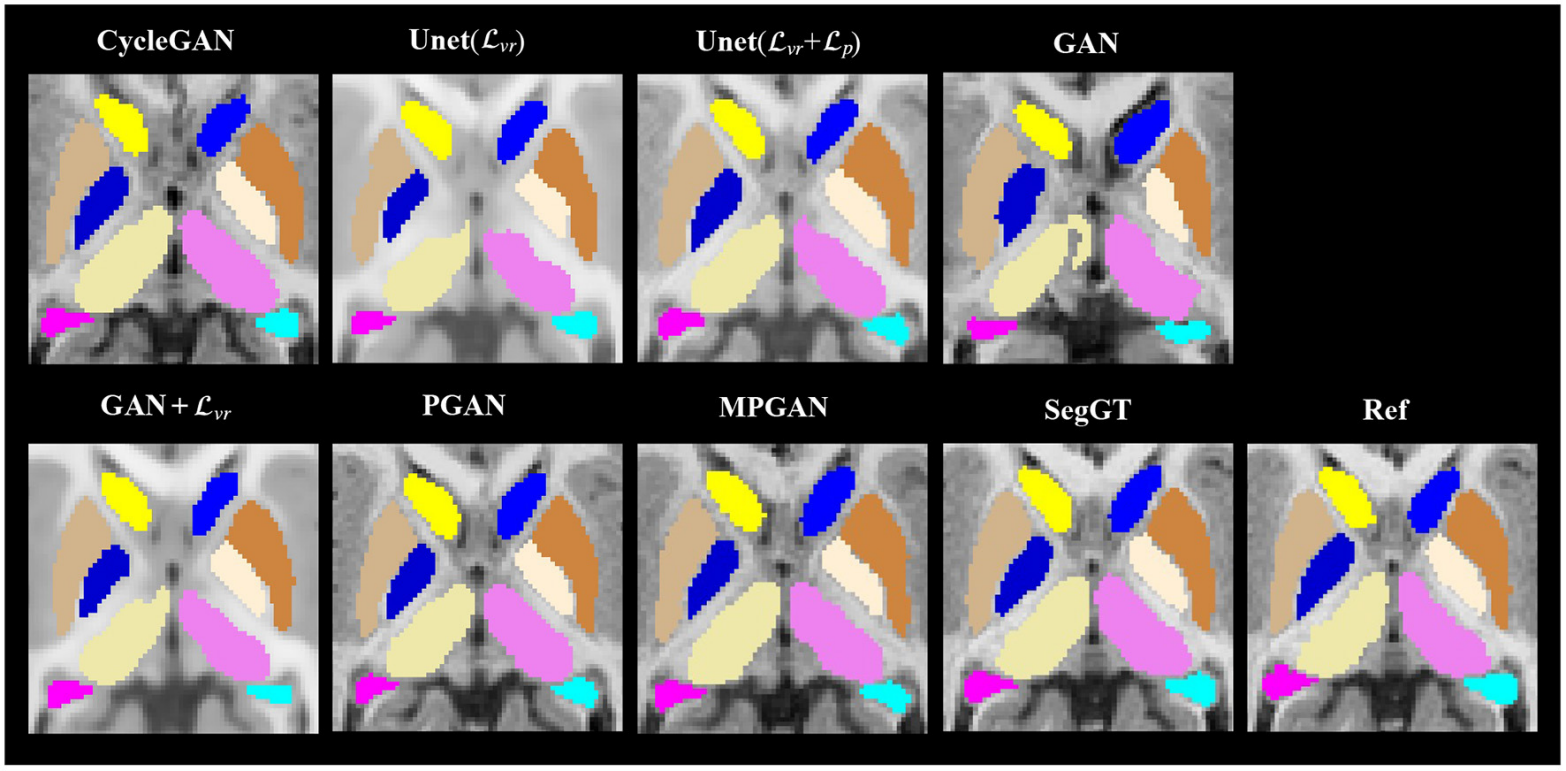
Examples of the subcortical segmentation results. The first four columns are the automatic segmentations of the predicted and the ground-truth images (SegGT). The last column is the reference manual segmentation (Ref).

In this section, we assess the quality of the predicted images in two segmentation tasks. We conducted subcortical and tissue segmentation on both predicted and ground-truth images at 12 months using an existing multi-atlas segmentation method (Wang et al., 2014). For the tissue segmentation task, the brain was segmented into four types of tissue, i.e., white matter, cortical gray matter, deep gray matter, and cerebrospinal fluid (CSF). For the subcortical segmentation task, 12 subcortical structure labels were computed: left and right hemispheric caudate, putamen, pallidum, thalamus, amygdala, and hippocampus. Examples of tissue and subcortical segmentation results are shown in Figures 5, 6, respectively. Our quantitative evaluation is based on following three metrics that measure the segmentation similarity between two segmentation results (*S*_1_ and *S*_2_): relative absolute volume difference (AVD, in %), average symmetric surface distance (ASD, in *mm*), and Dice coefficient. The relative absolute volume difference is as

(9) $$\begin{array}{l} AVD=\frac{\left|V_{S_{1}}-V_{S_{2}}\right|}{V_{S_{1}}}\times 100\% \end{array}$$

where *V*_*S*_1__ is the volume of *S*_1_ and *V*_*S*_2__ can be defined similarly. Suppose *B*_*S*_1__ and *B*_*S*_2__ are the borders of *S*_1_ and *S*_2_, respectively. The average symmetric surface distance (ASD) (Van Ginneken et al., 2007) is the mean of the closest distances from voxels on *B*_*S*_1__ to *B*_*S*_2__ and from voxels on *B*_*S*_2__ to *B*_*S*_1__, respectively. It can be defined as

(10) $$\begin{array}{l} ASD=\frac{1}{\left|B_{S_{1}}|+|B_{S_{2}}\right|}\times \left(\underset{x\in B_{S_{1}}}{\sum }d\left(x,B_{S_{2}}\right)+\underset{x\in B_{S_{2}}}{\sum }d\left(x,B_{S_{1}}\right)\right) \end{array}$$

The Dice coefficient evaluates the spatial overlap between two segmentation results, which is defined as

(11) $$\begin{array}{l} Dice=\frac{2|S_{1}\cap S_{2}|}{\left|S_{1}|+|S_{2}\right|} \end{array}$$

In order to obtain a overall evaluation criterion, we also combine the multiple metrics into a single fused score (FS). We follow (Van Ginneken et al., 2007) for the fused score, and thus use the *TanimotoError* as a measure of overlap instead of Dice coefficient when calculating FS. FS is formulated as

(12) $$\begin{array}{l} FS=\frac{1}{3}\left(\frac{AVD}{refAVD}+\frac{ASD}{refASD}+\frac{TanimotoError}{refTanimotoError}\right) \end{array}$$

where *TanimotoError* is

(13) $$\begin{array}{l} TanimotoError=\frac{\left|S_{1}\cup S_{2}|-|S_{1}\cap S_{2}\right|}{\left|S_{1}\cup S_{2}\right|} \end{array}$$

As in Van Ginneken et al. (2007), *refAVD*, *refASD*, and *refTanimotoError* are set to 5.6%, 0.27 mm, and 15.8%, respectively, based on the manual segmentation variance among human experts.

#### 3.5.1. Segmentation Consistency Analysis

In this section, we aim at assessing the quality of predicted images by evaluating how well the automatic segmentations of the predicted images match the ones of the ground-truth images. The intuition is that if two images are segmented by the same algorithm, the more similar the two images are, the more similar their segmentation results should be. The comparison results on subcortical segmentation task are presented in Table 3. We observe that, with respect to AVD, our PGAN and MPGAN significantly outperform all the other methods (*p* < 0.05) and MPGAN achieves the best performance. We can also see that, for ASD and Dice, there are no significant differences among Unet($L_{vr}$ + $L_{p}$), PGAN, and MPGAN, but our PGAN and MPGAN show a superior performance (*p* < 0.05) compared to the remaining four methods. While the images generated by Unet($L_{vr}$ + $L_{p}$) are visually of lower quality owing to blurred and unrealistic details (see Figures 2, 3), in this segmentation analysis, Unet($L_{vr}$ + $L_{p}$) performs at acceptable level for ASD and Dice coefficient. A possible explanation for this is that the segmentation algorithm we applied is robust to the image quality, to some extent, for this subcortical segmentation task. As for the fused score, our PGAN and MPGAN methods significantly outperform than other methods and MPGAN achieves the best score.

**Table 3:** Segmentation consistency across different approaches on subcortical segmentation task.

	**AVD ↓**	**ASD ↓**	**Dice ↑**	**FusedScore ↓**
SegPredict_GAN_ vs. SegGT	^*^^Δ^15.436 ± 7.683	^*^^Δ^0.800 ± 0.069	^*^^Δ^0.738 ± 0.077	^*^^Δ^2.760 ± 0.727
SegPredict_Unet(L_vr_)_ vs. SegGT	^*^^Δ^19.224 ± 7.934	^*^^Δ^0.672 ± 0.071	^*^^Δ^0.781 ± 0.058	^*^^Δ^2.712 ± 0.690
SegPredict_GAN+L_vr__ vs. SegGT	^*^^Δ^15.656 ± 5.545	^*^^Δ^0.632 ± 0.039	^*^^Δ^0.794 ± 0.052	^*^^Δ^2.415 ± 0.494
SegPredict_CycleGAN_ vs. SegGT	^*^^Δ^12.909 ± 4.947	^*^^Δ^0.678 ± 0.094	^*^^Δ^0.779 ± 0.057	^*^^Δ^2.345 ± 0.534
SegPredict_Unet(L_vr_+L_p_)_ vs. SegGT	^*^^Δ^7.566 ± 2.107	0.555 ± 0.021	0.820 ± 0.044	^*^^Δ^1.761 ± 0.261
SegPredict_PGAN_ vs. SegGT	^*^5.638 ± 2.049	0.555 ± 0.030	0.820 ± 0.047	^*^1.644 ± 0.274
SegPredict_MPGAN_ vs. SegGT	^Δ^ **5.153** **±** **1.767**	0.556 ± 0.025	0.820 ± 0.043	^Δ^ **1.618** **±** **0.235**

*SegPredict_CycleGAN_, SegPredict_Unet(L_vr_)_, SegPredict_Unet(L_vr_+L_p_)_, SegPredict_GAN_, SegPredict_GAN+L_vr__, SegPredict_PGAN_ and SegPredict_MPGAN_ denote automatic segmentations on the predicted images from CycleGAN, Unet($L_{vr}$), Unet($L_{vr}+L_{p}$), GAN, GAN+$L_{vr}$, PGAN, and MPGAN, respectively. SegGT is automatic segmentation on the ground-truth image*.

* *Significantly different compared to MPGAN (p < 0.05)*.

Δ *Significantly different compared to PGAN (p < 0.05)*.

*↓, lower is better. ↑, higher is better. The methods are sorted by the fused score. Methods with best performance are bolded for each metric (significantly better than other measurements, p < 0.5)*.

Table 4 lists the comparison results on the brain tissue segmentation task. The results confirmed the statistically significant better performance of our proposed methods (both PGAN and MPGAN) vs. the other methods, with respect to all metrics. The results clearly demonstrate the effectiveness of our proposed methods. Furthermore, MPGAN offers superior performance to PGAN in this task, which indicates that MPGAN benefits from using complementary information from multiple contrast data when performing a full brain tissue segmentation.

**Table 4:** Segmentation consistency across different approaches on tissue segmentation task.

	**AVD ↓**	**ASD ↓**	**Dice ↑**	**FusedScore ↓**
SegPredict_GAN_ vs. SegGT	^*^^Δ^4.556 ± 2.043	^*^^Δ^0.850 ± 0.148	^*^^Δ^0.709 ± 0.129	^*^^Δ^2.247 ± 0.451
SegPredict_CycleGAN_ vs. SegGT	^*^^Δ^6.486 ± 3.571	^*^^Δ^0.744 ± 0.101	^*^^Δ^0.739 ± 0.120	^*^^Δ^2.151 ± 0.399
SegPredict_Unet(L_vr_)_ vs. SegGT	^*^^Δ^6.538 ± 4.722	^*^^Δ^0.651 ± 0.052	^*^^Δ^0.777 ± 0.103	^*^^Δ^1.939 ± 0.408
SegPredict_GAN+L_vr__ vs. SegGT	^*^^Δ^4.797 ± 3.040	^*^^Δ^0.613 ± 0.041	^*^^Δ^0.779 ± 0.097	^*^^Δ^1.783 ± 0.285
SegPredict_Unet(L_vr_+L_p_)_ vs. SegGT	^*^^Δ^3.496 ± 1.263	^*^^Δ^0.591 ± 0.038	^*^^Δ^0.783 ± 0.104	^*^^Δ^1.668 ± 0.328
SegPredict_PGAN_ vs. SegGT	2.958 ± 1.566	^*^0.583 ± 0.038	^*^0.786 ± 0.108	^*^1.614 ± 0.384
SegPredict_MPGAN_ vs. SegGT	2.933 ± 1.404	^Δ^ **0.564** **±** **0.024**	^Δ^ **0.791** **±** **0.103**	^Δ^ **1.576** **±** **0.351**

*SegPredict_CycleGAN_, SegPredict_Unet(L_vr_)_, SegPredict_Unet(L_vr_+L_p_)_, SegPredict_GAN_, SegPredict_GAN+L_vr__, SegPredict_PGAN_ and SegPredict_MPGAN_ denote automatic segmentations on the predicted images from CycleGAN, Unet($L_{vr}$), Unet($L_{vr}+L_{p}$), GAN, GAN+$L_{vr}$, PGAN, and MPGAN, respectively. SegGT is automatic segmentation on the ground-truth image*.

* *Significantly different compared to MPGAN (p < 0.05)*.

Δ *Significantly different compared to PGAN (p < 0.05)*.

*↓, lower is better. ↑, higher is better. The methods are sorted by the fused score. Methods with best performance are bolded for each metric (significantly better than other measurements, p < 0.5)*.

#### 3.5.2. Segmentation Accuracy Analysis

In this section, we computed the AVD, ASD, and Dice coefficient between the reference manual segmentation (“Ref”) and the automatic segmentation of the ground-truth images or the predicted images. The values of these metrics here reflect the segmentation accuracy of the given image. Our goal is to evaluate the predicted images by comparing their segmentation accuracy with the one of the ground-truth image. Intuitively, the image which is more similar to the ground-truth image should have a closer segmentation accuracy to the ground-truth image. The segmentation accuracy comparison results on the subcortical segmentation task are shown in Table 5. We can observe that the images predicted by MPGAN achieve the closest AVD, Dice coefficient and fused score to the ground-truth images. Table 6 illustrates the segmentation accuracy comparison results on the tissue segmentation task. Similar to the subcortical segmentation task, MPGAN outperforms the other methods across most of the metrics (except AVD).

**Table 5:** Segmentation accuracy analysis on predicted images from different methods on subcortical segmentation task.

	**AVD ↓**	**ASD ↓**	**Dice ↑**	**FusedScore ↓**
SegPredict_Unet(L_vr_)_ vs. Ref	^*^^Δ^24.304 ± 9.173	^*^^Δ^0.832 ± 0.144	^*^^Δ^0.731 ± 0.085	^*^^Δ^3.349 ± 0.861
SegPredict_GAN_ vs. Ref	^*^^Δ^18.573 ± 10.597	^*^^Δ^0.940 ± 0.177	^*^^Δ^0.699 ± 0.096	^*^^Δ^3.218 ± 0.937
SegPredict_GAN+L_vr__ vs. Ref	^*^^Δ^20.715 ± 7.776	^*^^Δ^0.822 ± 0.122	^*^^Δ^0.737 ± 0.071	^*^^Δ^3.110 ± 0.670
SegPredict_CycleGAN_ vs. Ref	^*^^Δ^18.023 ± 6.998	^*^^Δ^0.749 ± 0.128	^*^^Δ^0.757 ± 0.060	^*^^Δ^2.807 ± 0.617
SegPredict_Unet(L_vr_+L_p_)_ vs. Ref	^*^^Δ^13.122 ± 5.901	^*^^Δ^0.748 ± 0.115	^*^^Δ^0.762 ± 0.060	^*^^Δ^2.502 ± 0.508
SegPredict_PGAN_ vs. Ref	^*^10.390 ± 6.217	0.720 ± 0.110	0.771 ± 0.056	^*^2.279 ± 0.505
SegPredict_MPGAN_ vs. Ref	^Δ^ **9.254** **±** **5.691**	0.721 ± 0.109	0.771 ± 0.051	^Δ^ **2.215** **±** **0.460**
SegGT vs. Ref	6.000 ± 4.396	0.666 ± 0.119	0.788 ± 0.055	1.902 ± 0.390

*SegPredict_CycleGAN_, SegPredict_Unet(L_vr_)_, SegPredict_Unet(L_vr_+L_p_)_, SegPredict_GAN_, SegPredict_GAN+L_vr__, SegPredict_PGAN_ and SegPredict_MPGAN_ denote automatic segmentations on the predicted images from CycleGAN, Unet($L_{vr}$), Unet($L_{vr}+L_{p}$), GAN, GAN+$L_{vr}$, PGAN, and MPGAN, respectively. SegGT is automatic segmentation on the ground-truth image and Ref denotes manual segmentation*.

* *Significantly different compared to MPGAN (p < 0.05)*.

Δ *Significantly different compared to PGAN (p < 0.05)*.

*The methods are sorted by the fused core. Methods with best performance are bolded for each metric (significantly better than other measurements, p < 0.5)*.

**Table 6:** Segmentation accuracy analysis on predicted images from different methods on tissue segmentation task.

	**AVD ↓**	**ASD ↓**	**Dice ↑**	**FusedScore ↓**
SegPredict_GAN_ vs. Ref	4.090 ± 2.507	^*^^Δ^0.904 ± 0.143	^*^^Δ^0.689 ± 0.151	^*^^Δ^2.346 ± 0.575
SegPredict_CycleGAN_ vs. Ref	5.493 ± 2.908	^*^^Δ^0.747 ± 0.093	^*^^Δ^0.724 ± 0.143	^*^^Δ^2.130 ± 0.458
SegPredict_Unet(L_vr_)_ vs. Ref	^*^^Δ^8.431 ± 5.839	^*^^Δ^0.687 ± 0.026	^*^^Δ^0.768 ± 0.123	^*^^Δ^2.120 ± 0.634
SegPredict_GAN+L_vr__ vs. Ref	^*^^Δ^5.302 ± 2.105	^*^^Δ^0.636 ± 0.024	^*^^Δ^0.776 ± 0.118	^*^^Δ^1.849 ± 0.384
SegPredict_Unet(L_vr_+L_p_)_ vs. Ref	3.610 ± 2.109	^*^0.589 ± 0.031	^*^0.784 ± 0.119	^*^1.667 ± 0.373
SegPredict_PGAN_ vs. Ref	3.817 ± 1.506	^*^0.583 ± 0.031	^*^0.788 ± 0.115	^*^1.662 ± 0.352
SegPredict_MPGAN_ vs. Ref	3.948 ± 1.285	^Δ^ **0.531** **±** **0.049**	^Δ^ **0.800** **±** **0.112**	^Δ^ **1.574** **±** **0.343**
SegGT vs. Ref	4.158 ± 2.604	0.376 ± 0.075	0.871 ± 0.058	1.185 ± 0.270

*SegPredict_CycleGAN_, SegPredict_Unet(L_vr_)_, SegPredict_Unet(L_vr_+L_p_)_, SegPredict_GAN_, SegPredict_GAN+L_vr__, SegPredict_PGAN_ and SegPredict_MPGAN_ denote automatic segmentations on the predicted images from CycleGAN, Unet($L_{vr}$), Unet($L_{vr}+L_{p}$), GAN, GAN+$L_{vr}$, PGAN, and MPGAN, respectively. SegGT is automatic segmentation on the ground-truth image and Ref denotes manual segmentation*.

* *Significantly different compared to MPGAN (p < 0.05)*.

Δ *Significantly different compared to PGAN (p < 0.05)*.

*The methods are sorted by the fused score. Methods with best performance are bolded for each metric (significantly better than other measurements, p < 0.5)*.

### 3.6. Efficacy of Data Imputation

The issue of missing scans is a common, practical problem in longitudinal studies. Subjects with incomplete scans cannot be used as training samples for machine learning applications as well as also for statistical methods that need complete data. Thus, the training size is significantly reduced due to these missing scans. Intuitively, using a larger training set is expected to improve performance, because adding training samples can bring more information and increase the diversity of the dataset. In this section, we use our method to predict missing subject scans for the purpose of machine learning tasks. After completing the data, these subjects can then be added to the training set for methods necessitating complete longitudinal subject data. While increasing the size of training samples via such imputation can improve the model performance, the imputed data has to be of high quality and representing the longitudinal data distribution appropriately. Poorly imputed data is expected to reduce model performance. Here, we investigated whether adding our imputed data to increase the size of the training set is beneficial to two practical tasks:

1. Classification of the severity group according to the social affect (SA) calibrated severity score (CSS) of the Autism Diagnostic Observation Schedule (ADOS, second edition) (Lord et al., 2012) at 24 months of age from prior longitudinal image data at 6 and 12 months
2. Regression of the gestational age at birth from later longitudinal image data at 6 and 12 months.

We compared the results of two settings: “non-imputed” and “imputed.” For the “non-imputed” setting, the classifier/regressor was trained with only real image pairs, i.e., both 6-month and 12-month images are real. For the “imputed” setting, in addition to the real training pairs used in the first setting, the classifier/regressor was also trained on “mixed” image pairs, i.e., real 6-month and predicted 12-month images, or predicted 6-month and real 12-month images. No imputed data was employed in the testing set. Thus, any differences in performance would stem from the additional inclusion of the imputed/generated datasets in the training set.

In our experiments, the real image pairs were divided into 4 folds and a 4-fold cross-validation was employed to evaluate the performance. Each time one fold was used for testing, and the other three were used for training. For the “imputed” setting, an additional set of “mixed” image pairs were included in all training folds of the cross-validation scheme. Since MPGAN performed the best in previous experiments, here we only employed MPGAN for image imputation. The Extreme Gradient Boosting (Xgboost) algorithm (Chen and Guestrin, 2016) was applied in both classification and regression tasks, which was implemented using the scikit-learn Python libraries (Raschka, 2015). Instead of directly feeding the raw images into the Xgboost model, which would result in an extremely high dimensional feature space, we employed the features extracted by the model genesis encoding (the 3D deep learning model previously used for the perceptual loss in section 2.4.1) as our inputs.

#### 3.6.1. ADOS-SA-CSS Group Classification at 24 Months With Imputed Data

Our goal in this experiment was to classify subject image data into one of three social affect severity groups (typical: score 1–2, low: score 3–4, moderate-to-high: score 5–10 Hus et al., 2014) at 24 months of age using 6 and 12 months MR image pairs. The ADOS-SA-CSS is a calibrated score that was developed to capture the severity of symptoms in social affect in children with ASD. We selected ADOS-SA-CSS as prediction measure instead of other calibrated ADOS scores (Restricted and Repetitive Behavior, RRB, and total severity score) as the ADOS-SA-CSS was observed to have a smoother distribution in our sample, as well as prior work indicate wide-scale associations with atypical social behavior in ASD (Sato and Uono, 2019).

**Figure 7:**
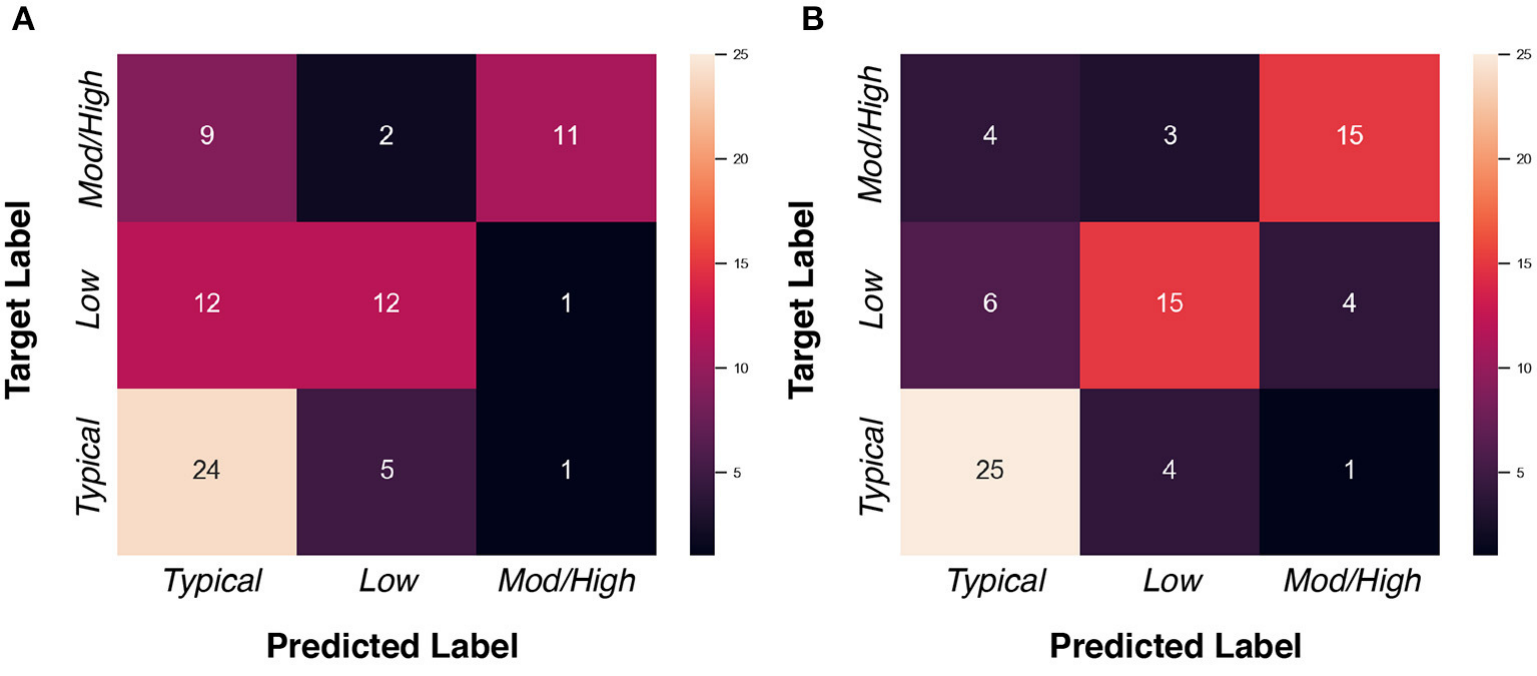
Confusion matrices of ADOS-SA-CSS group classification task. **(A)** “Non-imputed” setting. **(B)** “Imputed” setting.

We assessed whether data imputation improves the classification performance via F1 score, AUC score, and balanced accuracy metrics. Before data imputation, 77 subjects with complete scans at 6 and 12 month can be used for training. After data imputation, an additional 103 subjects can be added to the training set. Table 7 summarizes the cross-validated results in the “non-imputed” and “imputed” setting. We found that adding imputed data into training process can improve the model performance, such that the F1 score is increased from 0.590 to 0.694, the AUC score is increased from 0.655 to 0.671, and the balanced accuracy is increased from 0.594 to 0.699. We show the confusion matrices of the “non-imputed” and “imputed” setting in Figure 7. It is observed that the number of correctly classified samples is clearly improved across all groups, but especially in the moderate-to-high ADOS-SA-CSS group.

**Table 7:** Effects of imputed longitudinal data on the ADOS-SA-CSS group classification task.

**Setting**	***F*_1_Score ↑**	**AUC ↑**	**BalancedAccuracy ↑**
Non-imputed (77 subjects)	0.590	0.655	0.594
Imputed (77+103 subjects)	0.694	0.671	0.699

*↑, higher is better*.

#### 3.6.2. Regression of Gestational Age at Birth With Imputed Data

In this part, we employed the 6 and 12 months MR images to regress the gestational age at birth (39.03 ± 1.50 weeks). The mean absolute error (MAE) and relative error (RE) of the regressed GA were used as metrics. The 4-fold cross-validated results of “non-imputed” and “imputed” setting are shown in Table 8. Before data imputation, 134 subjects with complete scans at 6 and 12 month are used for training. After data imputation, an additional 76 subjects are added to the training set. Incorporating imputed data into the training process offers a slight improvement on the regression performance, though this improvement is of smaller magnitude than in the ADOS-SA-CSS classification.

**Table 8:** Effects of imputed longitudinal data on the gestational age (in weeks) regression task.

**Setting**	**RE ↓**	**MAE ↓**
Non-imputed (134 subjects)	2.559	0.971
Imputed (134 + 76 subjects)	2.327	0.886

*↓, lower is better*.

## 4. Discussion

In this paper, we present a novel adaptation of GAN to a new application: the longitudinal prediction of infant MR images. We validated and compared our technique with five alternative networks from multiple perspectives: qualitative and quantitative assessments of the image appearance, as well as quantitative assessment on two segmentation tasks. A consistent superior performance of our method has been shown in these evaluations, indicating its effectiveness. The images predicted by our method were then used for expanding the train set for ADOS-SA-CSS group classification and gestational age regression experiments. These experiments show that the imputed data brings a performance boost, highlighting the potential of our image prediction method when applied to a practical task.

Looking at Figures 2, 3, one can see that the 3D-Unet trained with only the voxel-wise reconstruction loss ($L_{vr}$) does a good job in keeping low-frequency information, and thus global structures are well-preserved. However, the images produced by it appear blurry and significantly lack high-frequency details, which is also reflected quantitatively with it achieving the worst LPIPS scores in Figure 4. We found that integrating the perceptual loss $L_{p}$ into the training can effectively alleviate this problem, as Unet($L_{vr}+L_{p}$) predicts sharper images, compared to Unet($L_{vr}$). Nevertheless, the images generated by Unet($L_{vr}+L_{p}$) still have unrealistic appearance from visual perspective. Using an adversarial training scheme, our proposed PGAN employs a voxel-wise reconstruction loss, a perceptual loss, and an adversarial loss jointly to produce sharper and more realistic images. This results in a statistically significant improvement in the quantitative assessment (see Figure 4). This may be due to the following reason: with respect to the adversarial learning strategy, the discriminator is optimized to differentiate the real and fake images. In order to fool the discriminator, the generator has to push the output distribution closer to the distribution of real data. As a result, the outputs of the generator are visually realistic.

As T1w and T2w images encompass rich information that is different and complementary to each other, we further propose a multi-contrast version, called MPGAN, which produces even finer details as well as achieves a better quantitative score, compared to PGAN. In particular, we observe a loss in the cortical contrast information in T2w images in most of evaluated methods, while it appears well-preserved for MPGAN. In summary, the combination of the voxel-wise reconstruction loss, the perceptual loss, the adversarial loss and the use of multi-contrast information allows our MPGAN to produce realistic images with accurate details where both low and high frequency information is well-preserved.

Also, we report analyses of the segmentation consistency and accuracy for the different methods in both subcortical and tissue segmentation tasks. As shown in Tables 3, 4, our MPGAN achieves the best segmentation consistency across most of the used metrics, where the differences are significant at the *p* < 0.05 level. The second-best performance is yielded by our single contrast PGAN method. As shown in Tables 5, 6, our MPGAN results in the closest segmentation accuracy to the ground truth images across most of the considered metrics. Besides, we also found that, with respect to ASD and Dice coefficient, there is no significant difference between PGAN and MPGAN for both segmentation consistency and accuracy analysis on the subcortical segmentation task. However, MPGAN clearly performs better than PGAN for tissue segmentation task. This may be attributed to the reason that subcortical segmentation is relatively simple because of consistent shape of subcortical structures, compared to the folded, complex cortex assessed in the tissue segmentation task.

To investigate the applicability of our predicted/imputed images, we employed our predicted image data for data imputation in two practical tasks, i.e., one on ADOS-SA-CSS group classification and the other on regression of gestational age. The results show that the model performance can be boosted with the help of imputed data for the both tasks. We did not employ the imputed data for testing purposes, so all gains in classification/regression are due to the inclusion of the imputed data. This finding indicates that the predicted data was sufficiently close to the true data that it provided valuable information to the training process.

It is further noteworthy that any image data prediction is biased by the training data. Thus, we expect our method to potentially perform poorly for brain images with atypical morphometry or neuropathology that are unknown to the trained model as such data was not included in the training. This indicates the necessity to develop additional safeguards to ensure that the input data and the trained model are appropriately matched. In the results presented here, we apply our methods in a fairly narrow subject population (typically developing children and children at familial risk for ASD) and all MRI data was inspected by a neuroradiologist for the presence of visible neuropathology.

While our method shows very promising results, it is not without limitations. For one, the current approach needs paired longitudinal data of the same subject for training. A further computation limitation is that we are directly feeding 3D data into our networks, which requires large amounts of memory and thus a high performance GPU server.

## 5. Conclusion

This paper introduces a novel multi-contrast perceptual generative network (MPGAN) for longitudinal prediction of infant MRI data. To the best of our knowledge, this is the first time that deep generative methods are applied for longitudinal prediction of structural MRI in the first year of life. Our approach improves the realism, sharpness, and accuracy of predicted images by merging the adversarial learning scheme with the voxel-wise reconstruction loss and the perceptual loss, as well as taking the multi-contrast information into account. In our qualitative and quantitative assessments, our method yielded a better performance than the alternative approaches studied in this work.

Longitudinal data is crucial to capture appropriate developmental trajectories in studies of the first year of life. Missing data is a major issue and our proposed method achieves highly promising results to impute such missing data for training data augmentation in classification or regression tasks. The improvement in performance when classifying subjects into categories of severity of social affect symptoms from image data only is quite impressive.

Our future work will focus on extending the paired approach at consistent time points (here 6 and 12 months of age) to a time regression based approach to overcome our current limitation of discrete time points and model imputation along the full first year of life. Furthermore, additional experiments to quantify the value of adding real data (i.e., acquiring additional subjects) vs. adding imputed data (i.e., imputing incomplete data as performed here) will need to be performed.

## Data Availability Statement

Code: The source code of our MPGAN method is available as open source at https://github.com/liying-peng/MPGAN. Data: All raw MRI datasets and associated demographic information employed in this study is available at NIH/NDA: https://nda.nih.gov/edit_collection.html?id=19.

## Ethics Statement

This study was reviewed and approved by the Institutional Review Boards (IRB) of all data collection sites (University of North 1654 Carolina, University of Washington, Children's Hospital of 1655 Pennsylvania, Washington University). Written informed consent was provided by the parent/guardian of all enrolled subjects. The data used in this work is collected from the Infant Brain Imaging Study (IBIS) database (https://www.ibis-network.org).

## Author Contributions

LP, LL, YL, Y-wC, GG, and MAS contributed to methodological development. LP, MAS, HH, CB, RG, MDS, and JG contributed to the study design. ACE, SD, AME, RM, KB, RS, HH, and JP contributed to the image data collection. LP, ZM, RV, SK, and MAS contributed to the experiment data collection. LP and MAS contributed to the statistical analysis. LP and MAS wrote the first draft of the manuscript. All authors contributed to manuscript revision, read, and approved the submitted version.

## Funding

This study was supported by grants from the Major Scientific Project of Zhejiang Lab (No. 2018DG0ZX01), the National Institutes of Health (R01-HD055741, T32-HD040127, U54-HD079124, U54-HD086984, R01-EB021391, and P50-HD103573), Autism Speaks, and the Simons Foundation (140209). MDS was supported by NIH career development award K12-HD001441, as is JG K01-MH122779. The sponsors had no role in the design and conduct of the study; collection, management, analysis, and interpretation of the data; preparation, review, or approval of the manuscript; and decision to submit the manuscript for publication.

## Conflict of Interest

The authors declare that the research was conducted in the absence of any commercial or financial relationships that could be construed as a potential conflict of interest.

## Publisher's Note

All claims expressed in this article are solely those of the authors and do not necessarily represent those of their affiliated organizations, or those of the publisher, the editors and the reviewers. Any product that may be evaluated in this article, or claim that may be made by its manufacturer, is not guaranteed or endorsed by the publisher.
