# Supplementary material for: Longitudinal Prediction of Infant MR Images With Multi-Contrast Perceptual Adversarial Learning
Source: Front Neurosci. 2021 Sep 9;15:653213. doi: 10.3389/fnins.2021.653213 (PMC8458966; doi:10.3389/fnins.2021.653213)
Supplement: Supplementary file 1 [file Data_Sheet_1.PDF]

Supplementary Material

1 PARAMETER OPTIMIZATION

In this section, we describe the selection of trade-off parameters α, β and the feature map $\phi_m(x)$ used for perceptual loss computation. α is the weight of the voxel reconstruction loss L_{vr}, β is the weight of the perceptual loss L_p and $\phi_m(x)$ is the output from the m-th layer of the feature extractor when processing the image x. As shown in Table S1, we first set α to zero and compare performance at different β and $\phi_m(x)$. After identifying the optimal β and $\phi_m(x)$, we fixed β and $\phi_m(x)$ and then compared performance at various values of α (see Table S1). The corresponding results show that α= 25, β= 25, $\phi_1(x)$ generates the best result.

Table S1. LPIPS for different β and $\phi_m(x)$. $\phi_1(x)$, $\phi_2(x)$, $\phi_3(x)$, and $\phi_4(x)$ denote the feature maps from the 1-st, 2-nd, 3-rd, and 4-th layer of the feature extractor, respectively. The results are for α = 0.

	β=5	β=15	β=25	β=35	β=45
$\phi_1(x)$	0.0420±0.0055	0.0421±0.0056	**0.0410±0.0057**	0.0415±0.0056	0.0412±0.0060
$\phi_2(x)$	0.0472±0.0064	0.0414±0.0054	0.0451±0.0052	0.0449±0.0054	0.0440±0.0057
$\phi_3(x)$	0.0480±0.0050	0.0467±0.0052	0.0656±0.0075	0.0602±0.0053	0.0618±0.0056
$\phi_4(x)$	0.0553±0.0046	0.0661±0.0057	0.0672±0.0055	0.0635±0.0066	0.0648±0.0068

Table S2. LPIPS for different α.

	α=5	α=15	α=25	α=35	α=45
β=25,$\phi_1(x)$	0.0409±0.0059	0.0403±0.0059	**0.0402±0.0057**	0.0403±0.0059	0.0415±0.0066
