# Supplementary figures and images for: Longitudinal Prediction of Infant MR Images With Multi-Contrast Perceptual Adversarial Learning
Source: Front Neurosci. 2021 Sep 9;15:653213. doi: 10.3389/fnins.2021.653213 (PMC8458966; doi:10.3389/fnins.2021.653213)

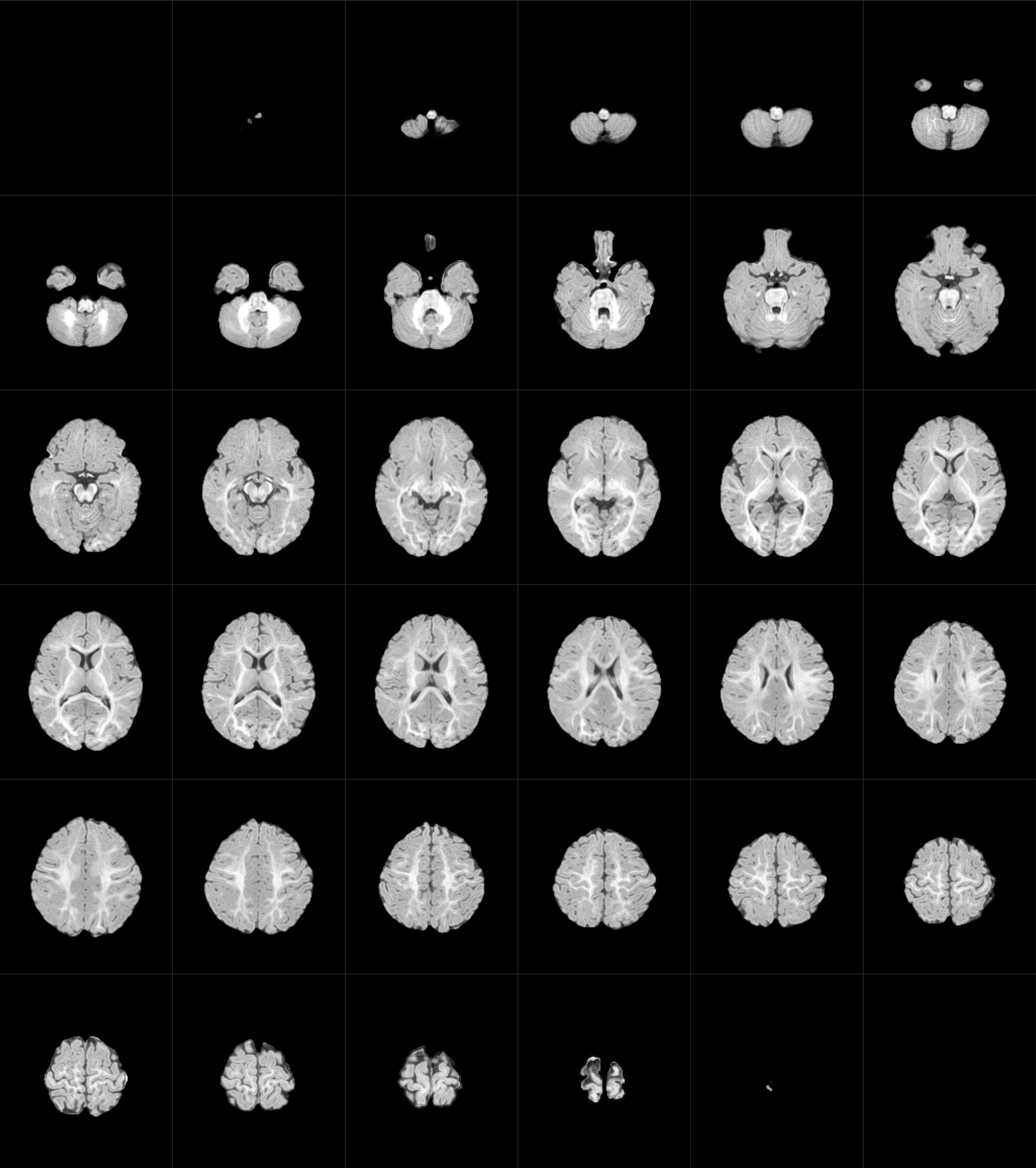

Supplement: Supplementary file 2 [file Data_Sheet_2.ZIP › 6monthFrom12MonthT1/6monthFrom12MonthT1_MPGAN.png]

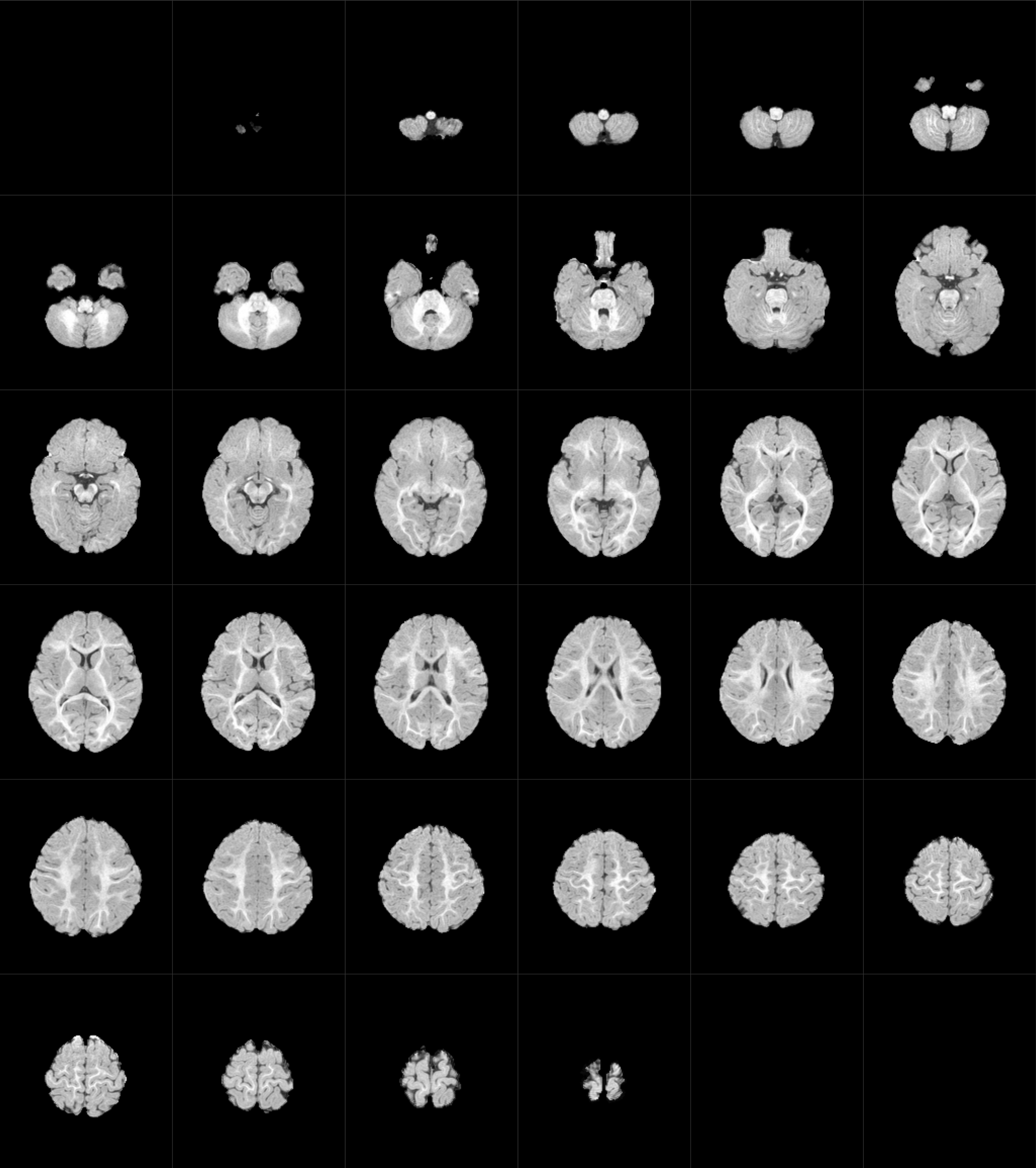

Supplement: Supplementary file 2 [file Data_Sheet_2.ZIP › 6monthFrom12MonthT1/6monthFrom12MonthT1_GroundTruth.png]

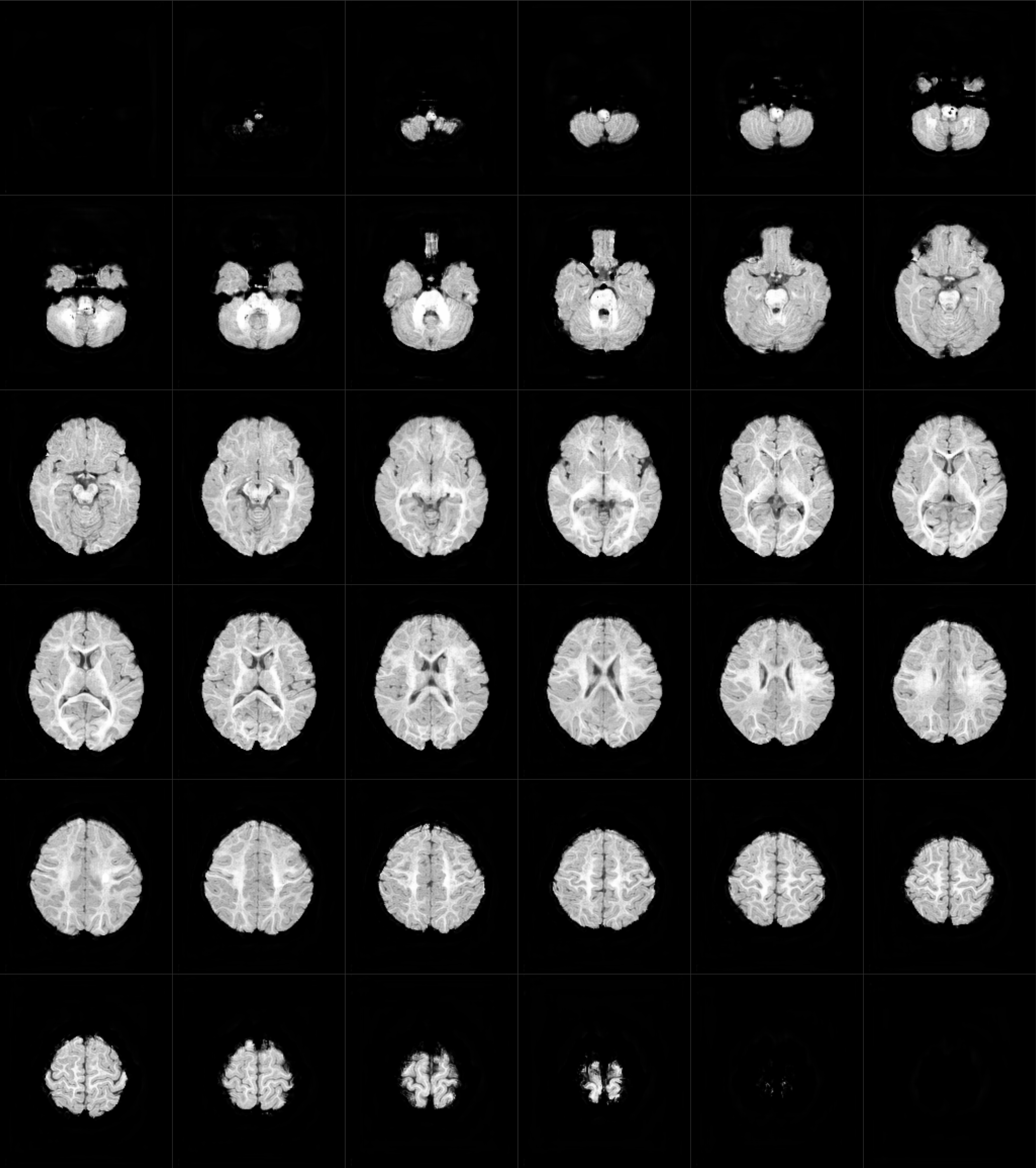

Supplement: Supplementary file 2 [file Data_Sheet_2.ZIP › 6monthFrom12MonthT1/6monthFrom12MonthT1_CycleGAN.png]

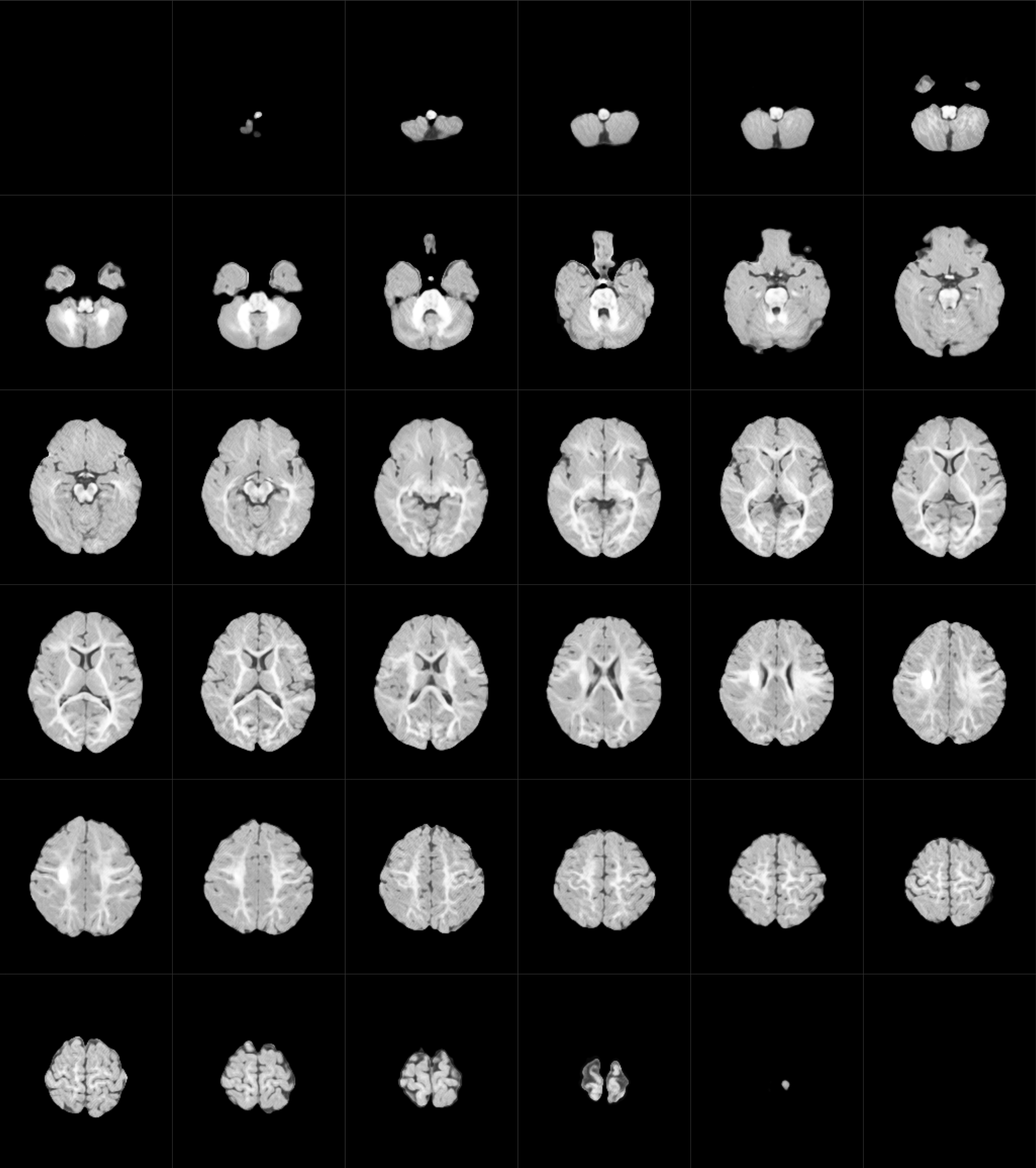

Supplement: Supplementary file 2 [file Data_Sheet_2.ZIP › 6monthFrom12MonthT1/6monthFrom12MonthT1_Unet_Lp.png]

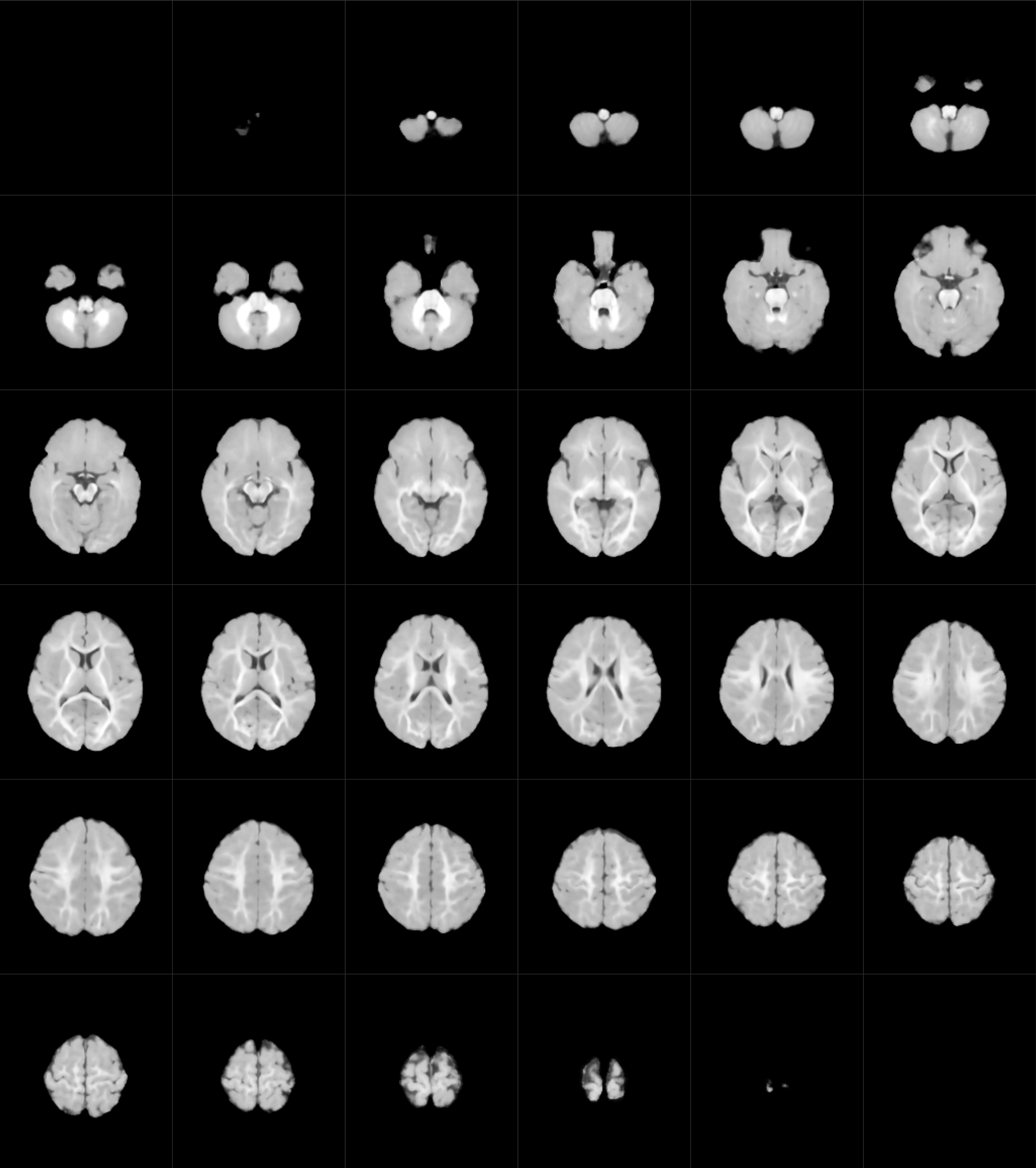

Supplement: Supplementary file 2 [file Data_Sheet_2.ZIP › 6monthFrom12MonthT1/6monthFrom12MonthT1_GAN_L1.png]

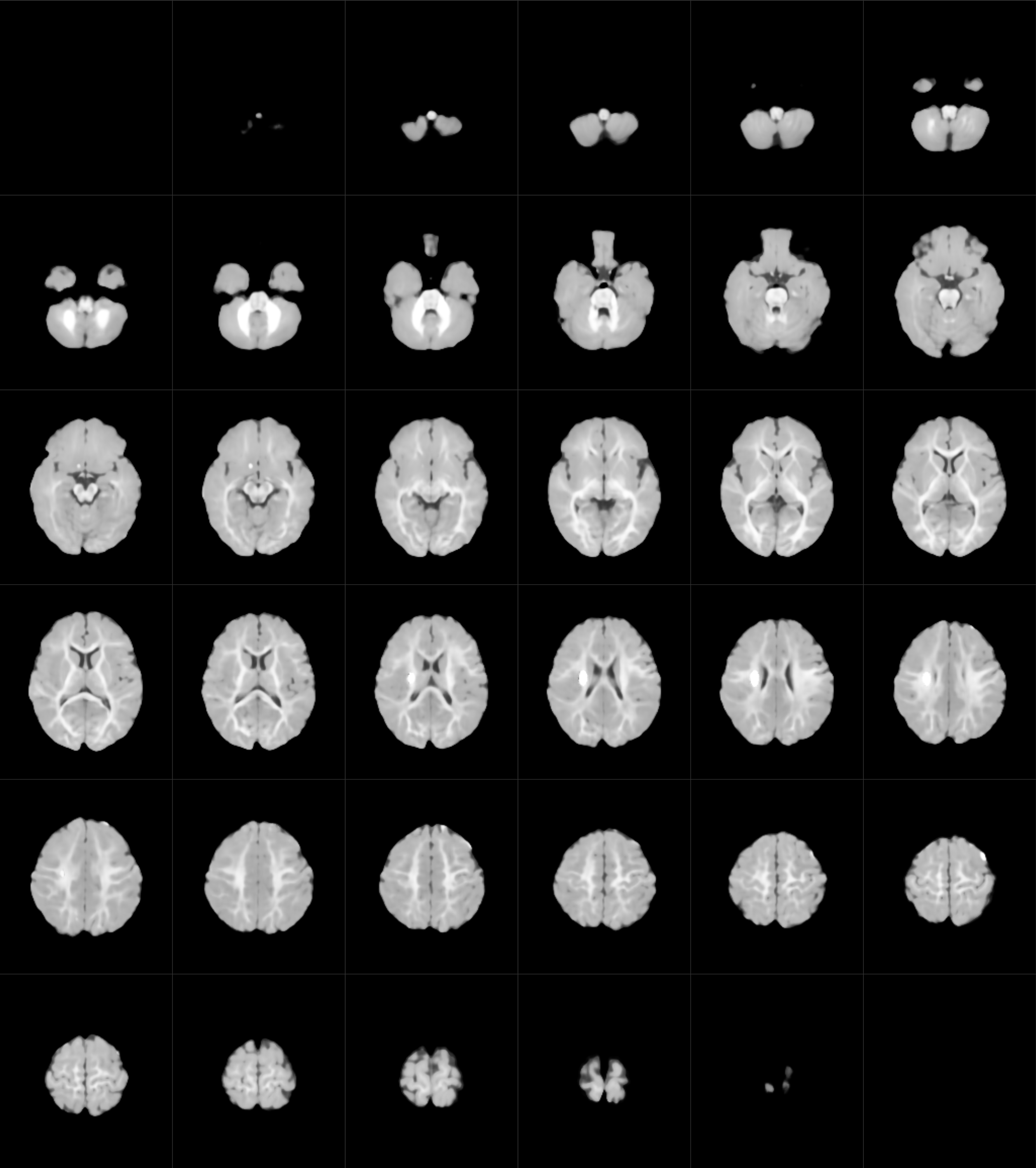

Supplement: Supplementary file 2 [file Data_Sheet_2.ZIP › 6monthFrom12MonthT1/6monthFrom12MonthT1_Unet.png]

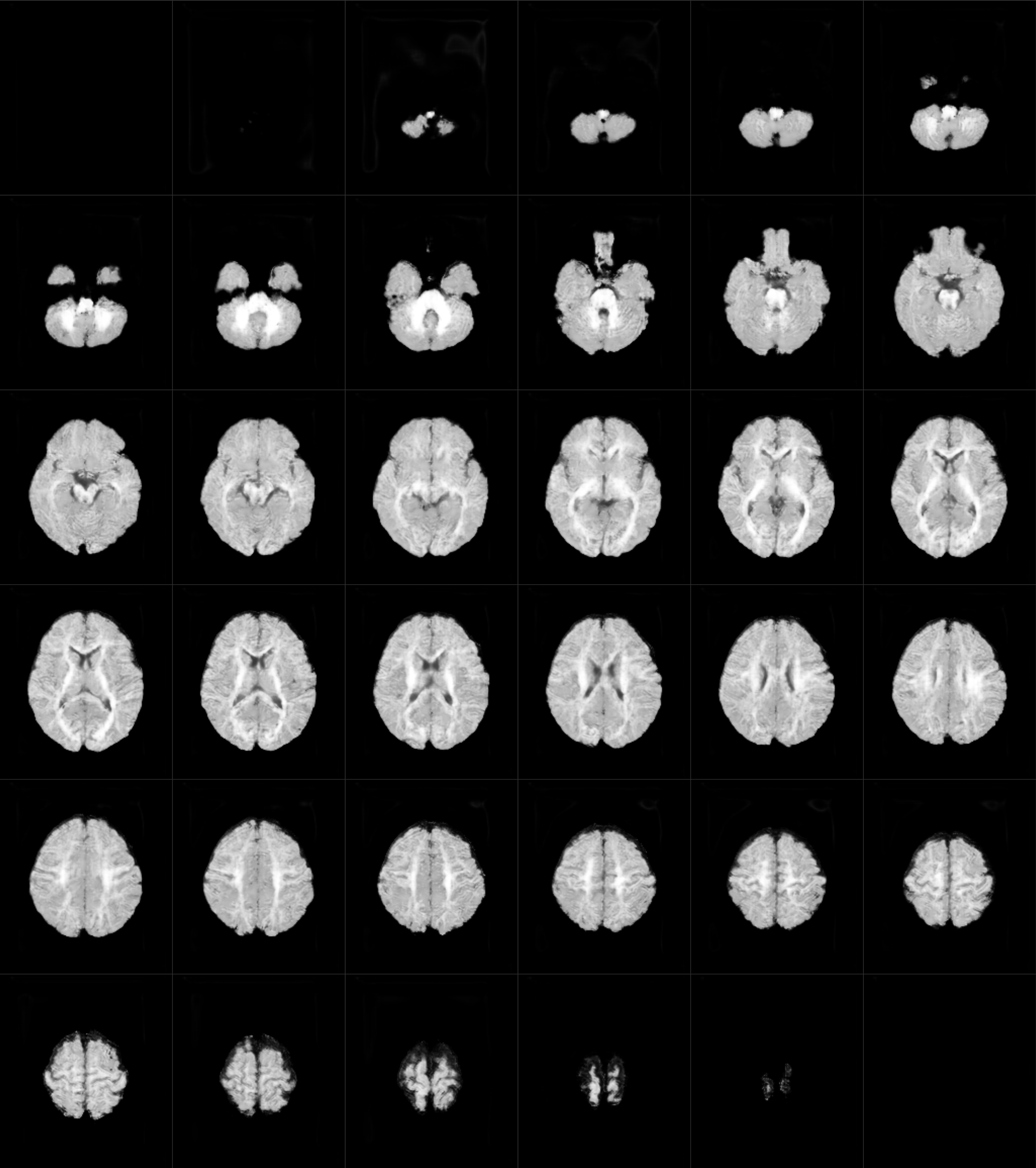

Supplement: Supplementary file 2 [file Data_Sheet_2.ZIP › 6monthFrom12MonthT1/6monthFrom12MonthT1_GAN.png]

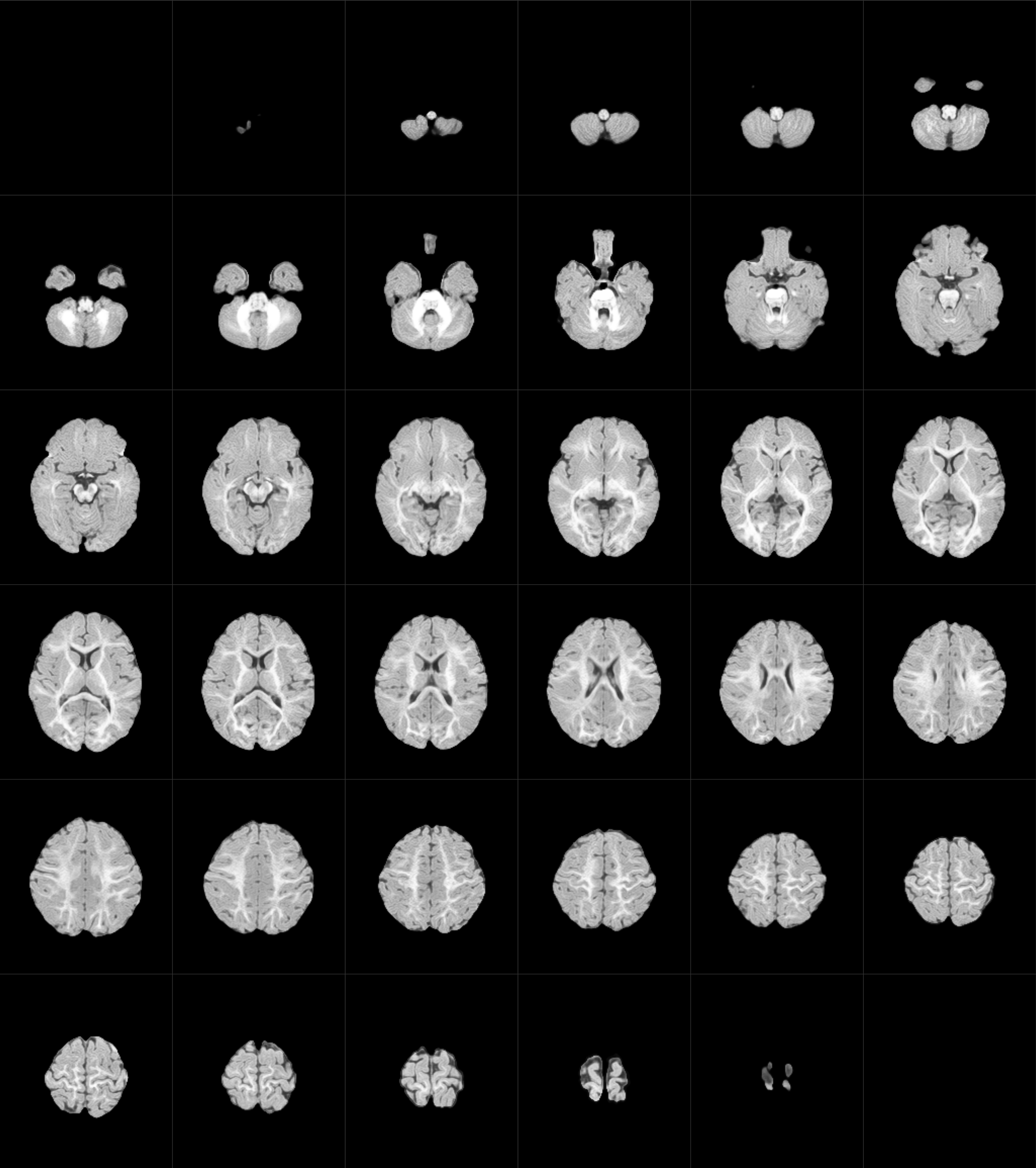

Supplement: Supplementary file 2 [file Data_Sheet_2.ZIP › 6monthFrom12MonthT1/6monthFrom12MonthT1_PGAN.png]

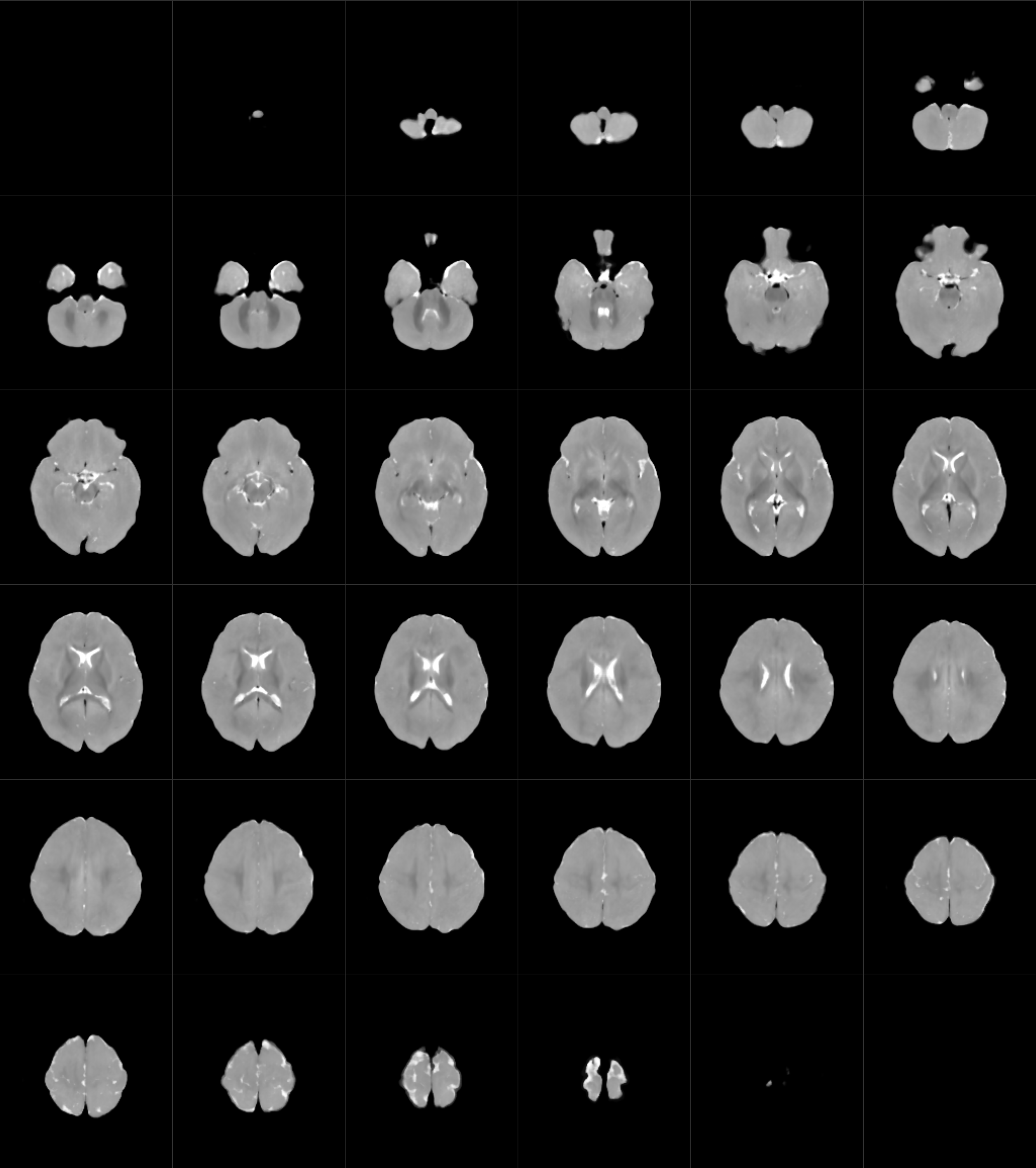

Supplement: Supplementary file 3 [file Data_Sheet_3.ZIP › 6monthFrom12MonthT2/6monthFrom12MonthT2_GAN_L1.png]

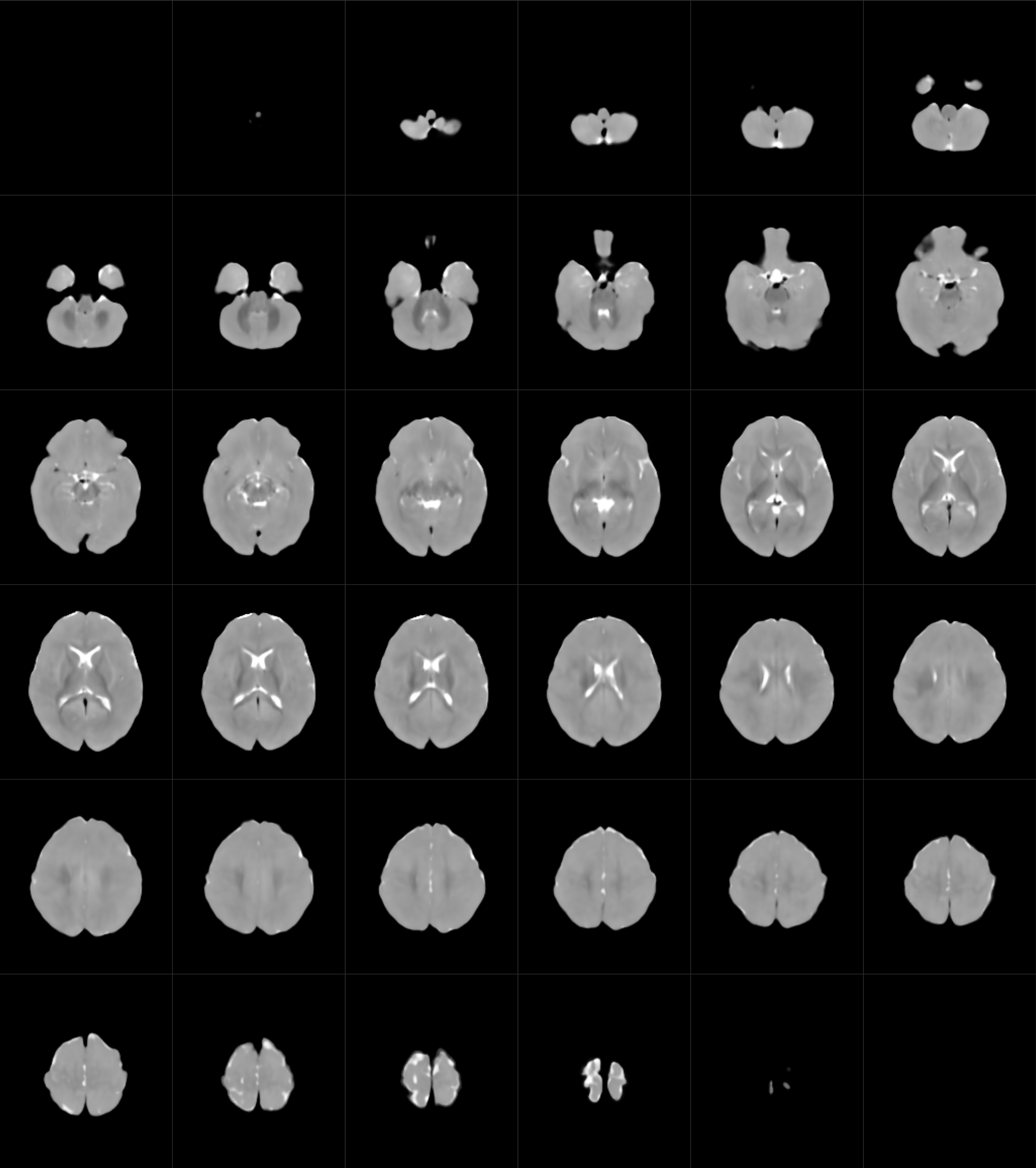

Supplement: Supplementary file 3 [file Data_Sheet_3.ZIP › 6monthFrom12MonthT2/6monthFrom12MonthT2_Unet.png]

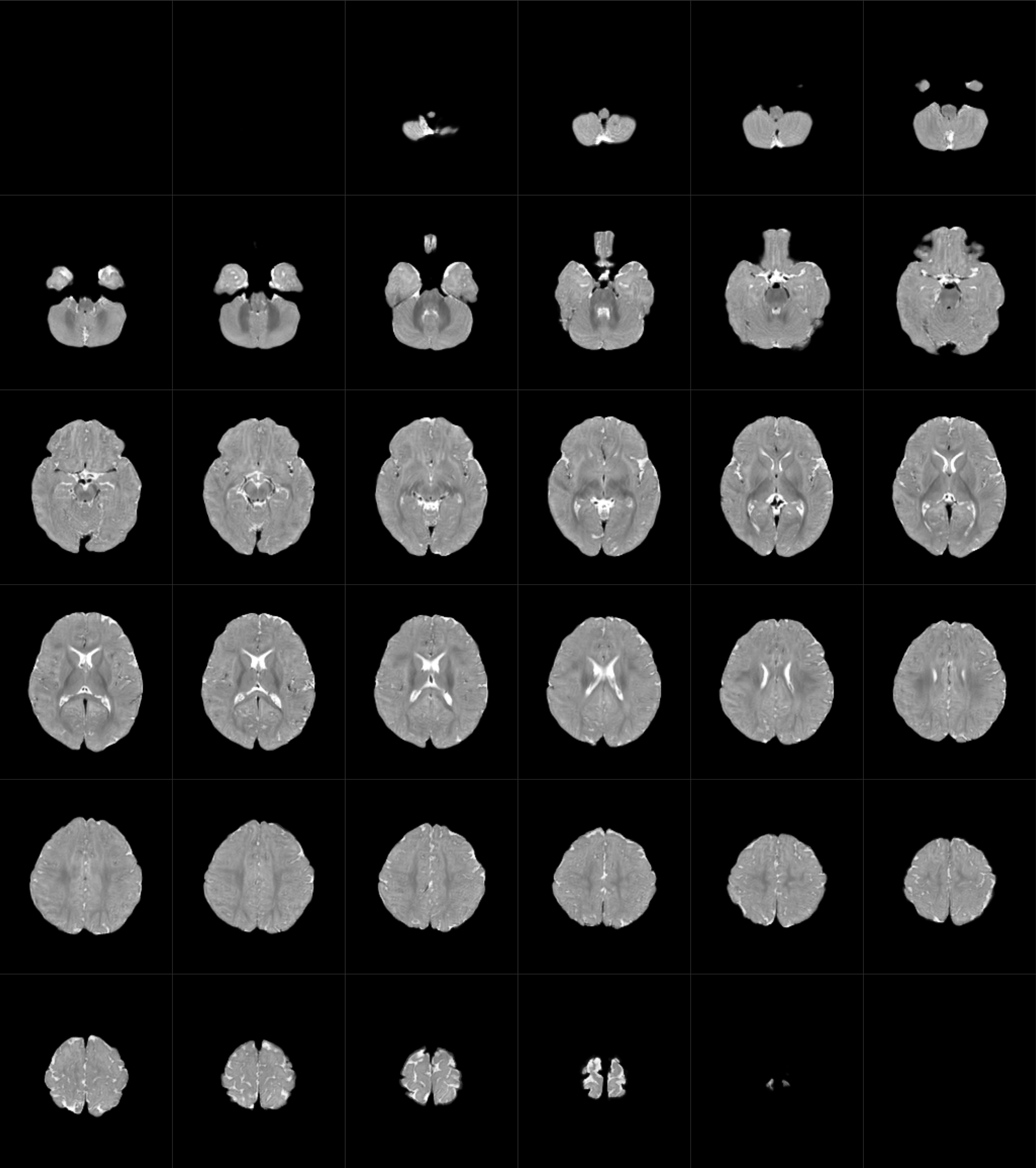

Supplement: Supplementary file 3 [file Data_Sheet_3.ZIP › 6monthFrom12MonthT2/6monthFrom12MonthT2_PGAN.png]

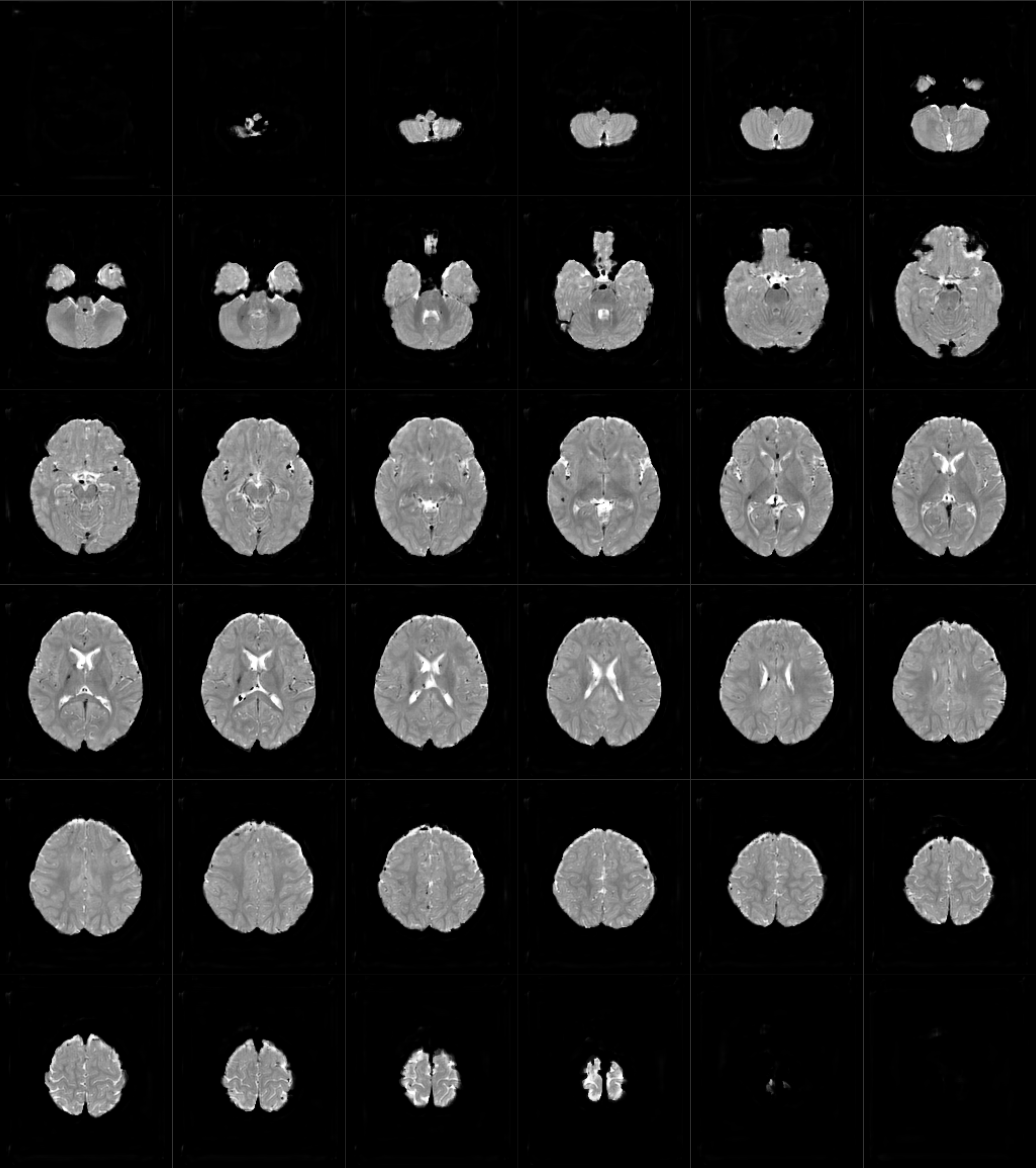

Supplement: Supplementary file 3 [file Data_Sheet_3.ZIP › 6monthFrom12MonthT2/6monthFrom12MonthT2_CycleGAN.png]

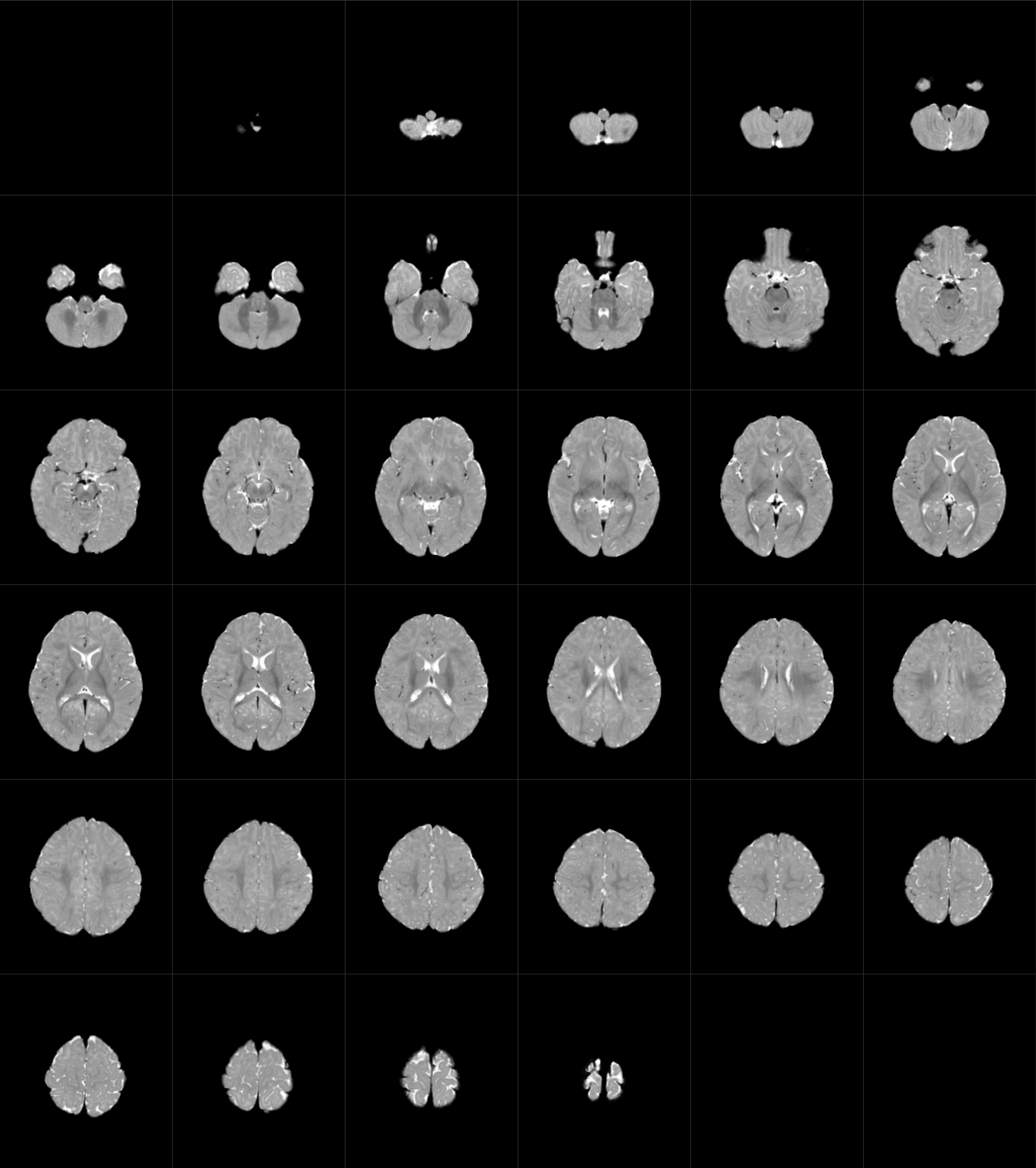

Supplement: Supplementary file 3 [file Data_Sheet_3.ZIP › 6monthFrom12MonthT2/6monthFrom12MonthT2_GroundTruth.png]

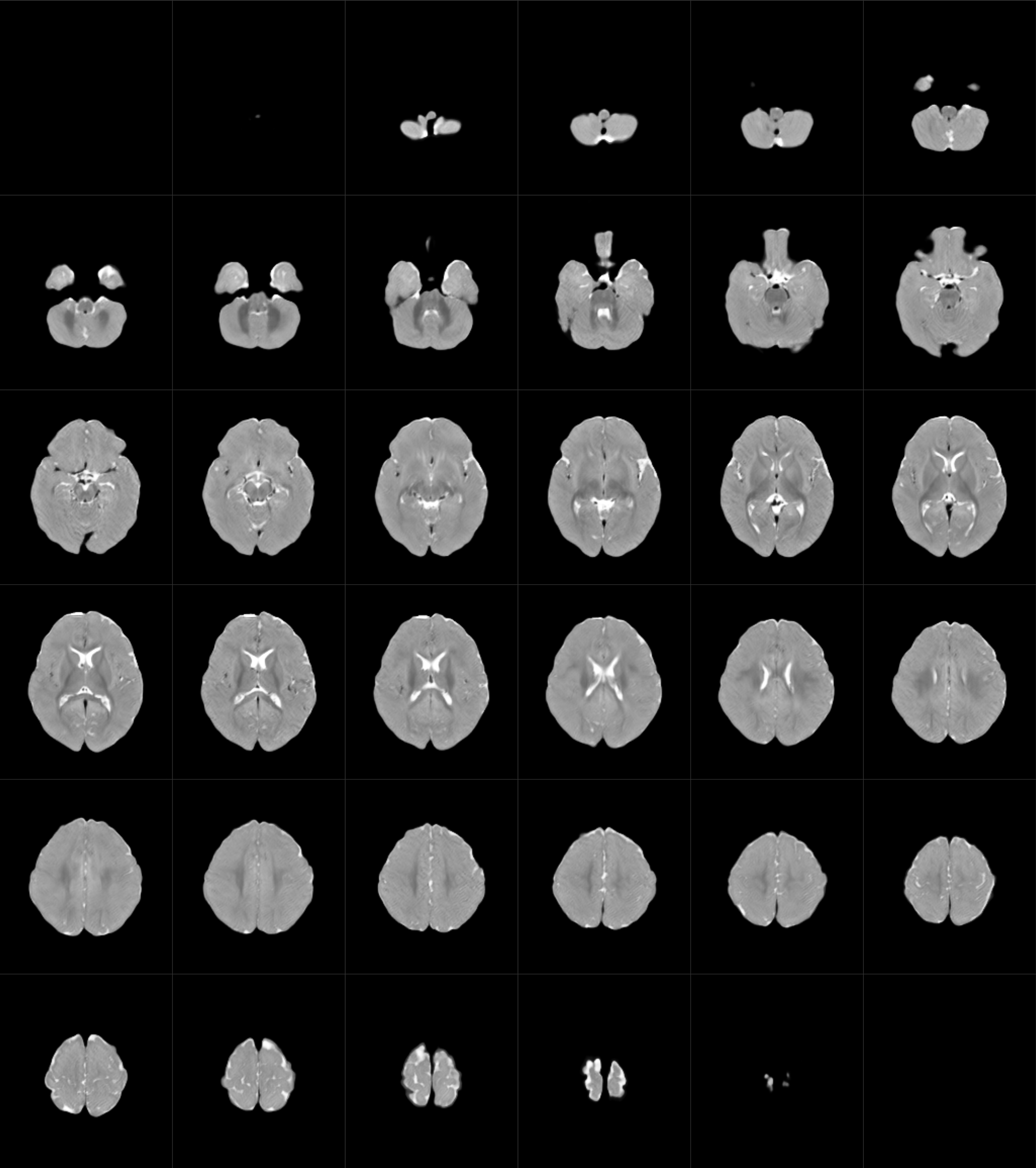

Supplement: Supplementary file 3 [file Data_Sheet_3.ZIP › 6monthFrom12MonthT2/6monthFrom12MonthT2_Unet_Lp.png]

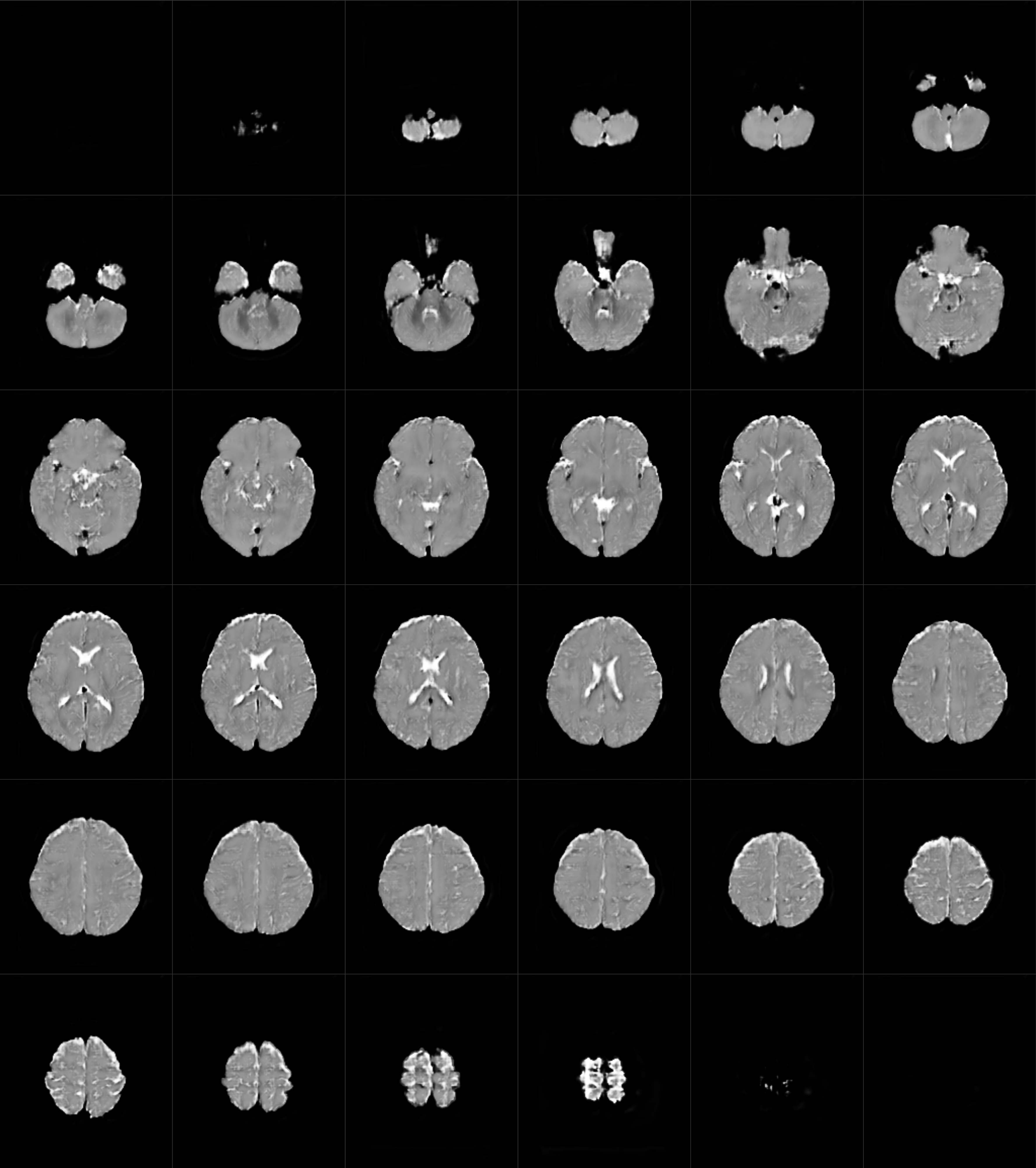

Supplement: Supplementary file 3 [file Data_Sheet_3.ZIP › 6monthFrom12MonthT2/6monthFrom12MonthT2_GAN.png]

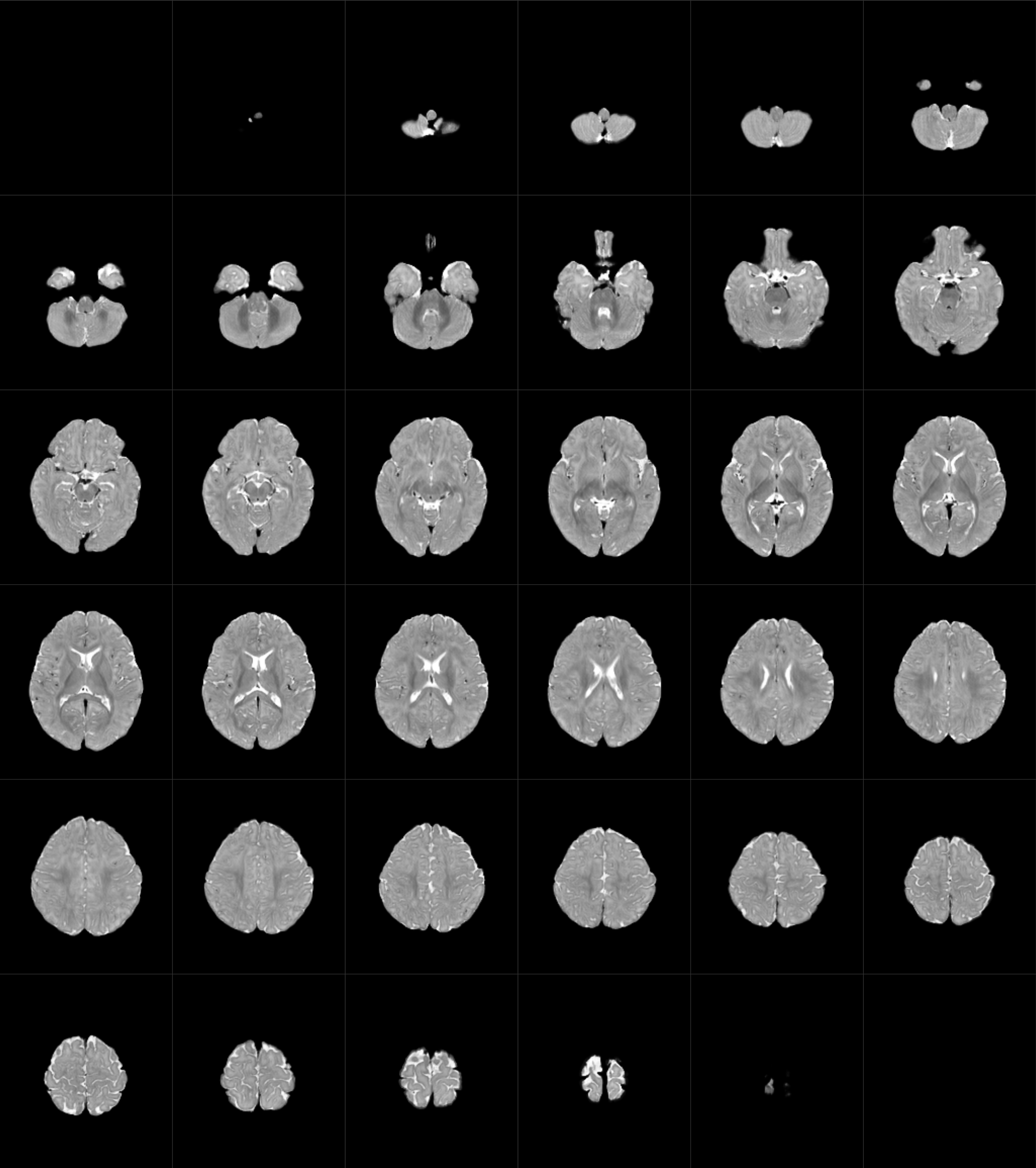

Supplement: Supplementary file 3 [file Data_Sheet_3.ZIP › 6monthFrom12MonthT2/6monthFrom12MonthT2_MPGAN.png]

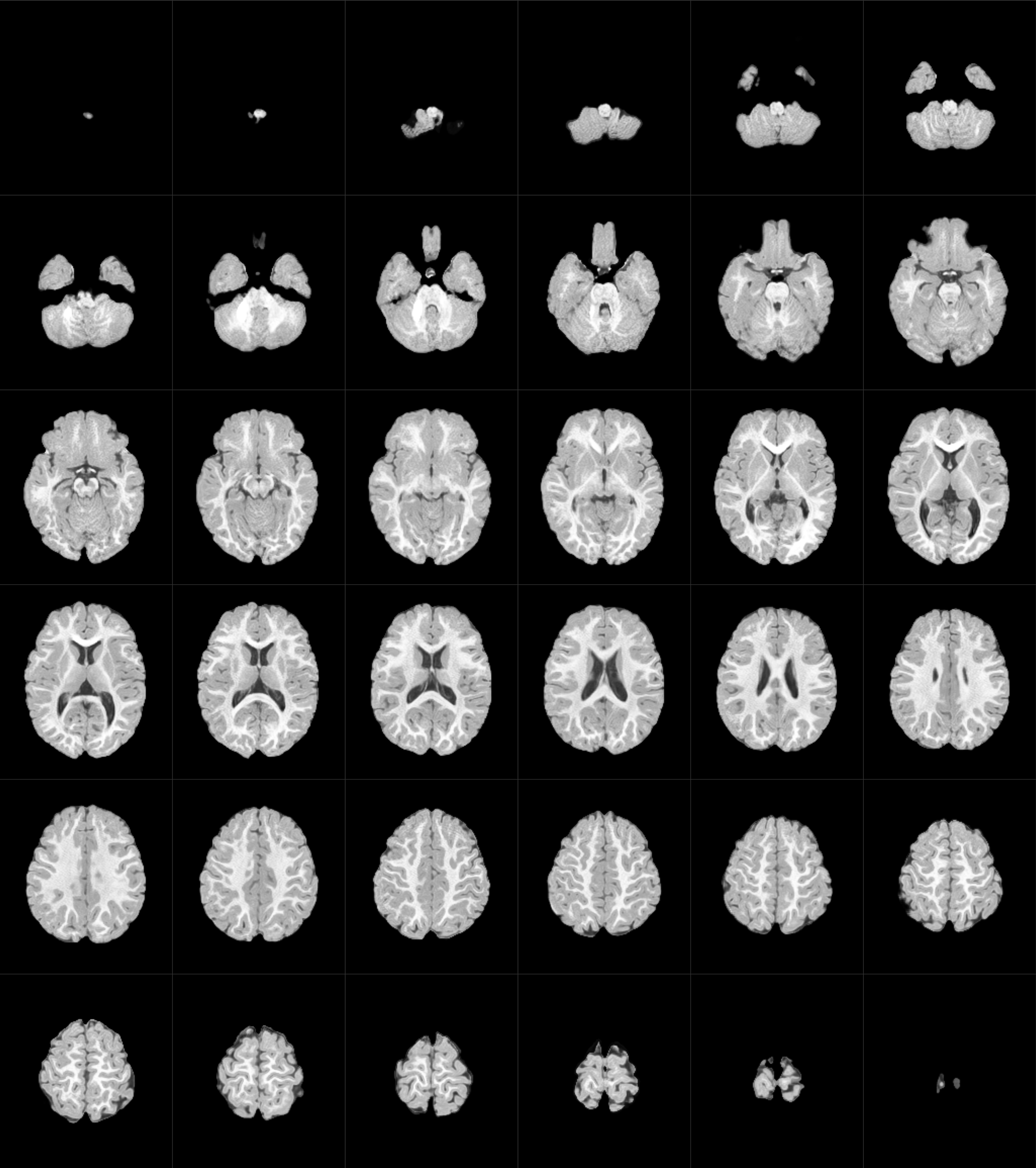

Supplement: Supplementary file 4 [file Data_Sheet_4.ZIP › 12monthFrom6MonthT1/12monthFrom6MonthT1_PGAN.png]

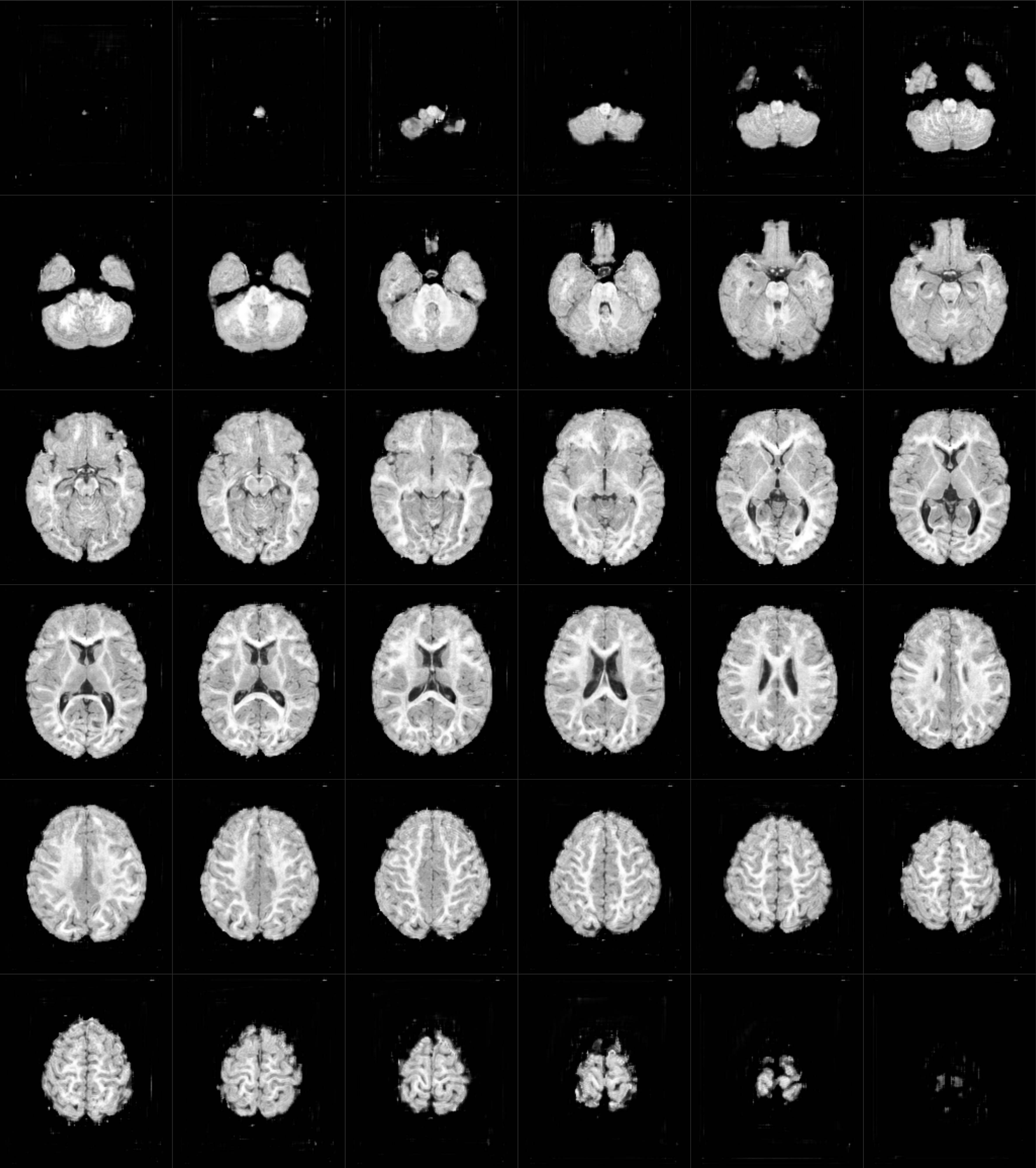

Supplement: Supplementary file 4 [file Data_Sheet_4.ZIP › 12monthFrom6MonthT1/12monthFrom6MonthT1_CycleGAN.png]

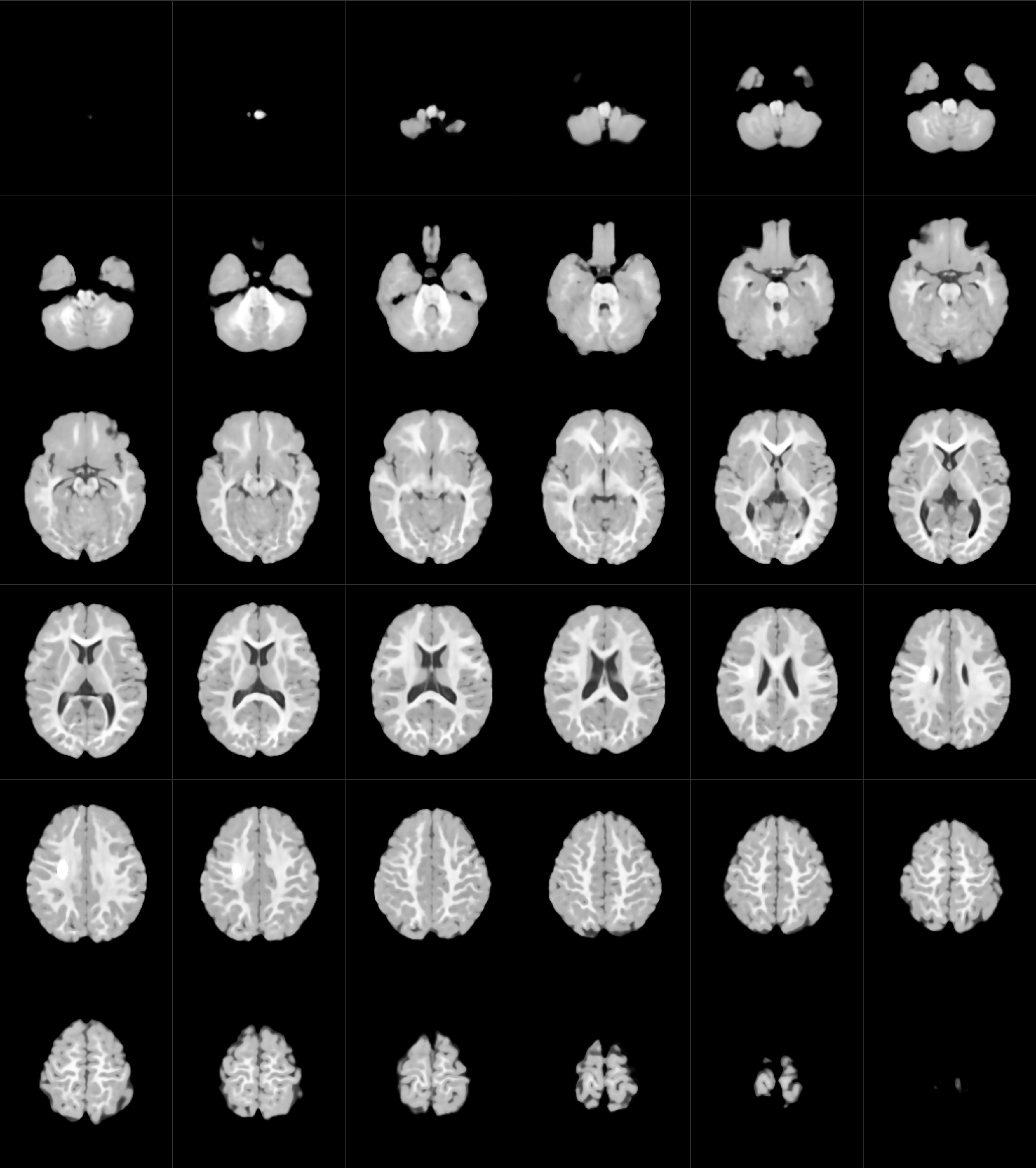

Supplement: Supplementary file 4 [file Data_Sheet_4.ZIP › 12monthFrom6MonthT1/12monthFrom6MonthT1_Unet.png]

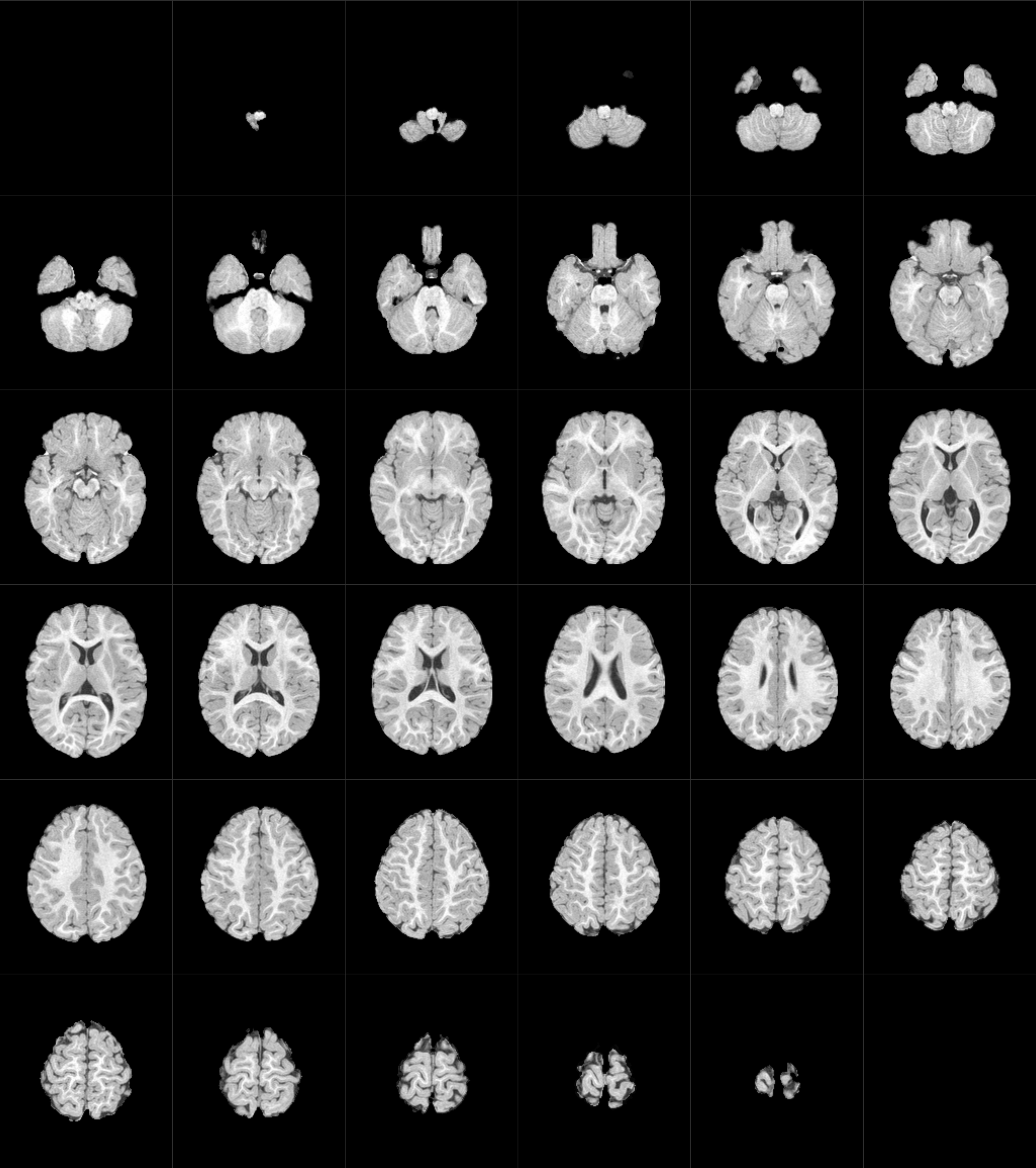

Supplement: Supplementary file 4 [file Data_Sheet_4.ZIP › 12monthFrom6MonthT1/12monthFrom6MonthT1_GroundTruth.png]

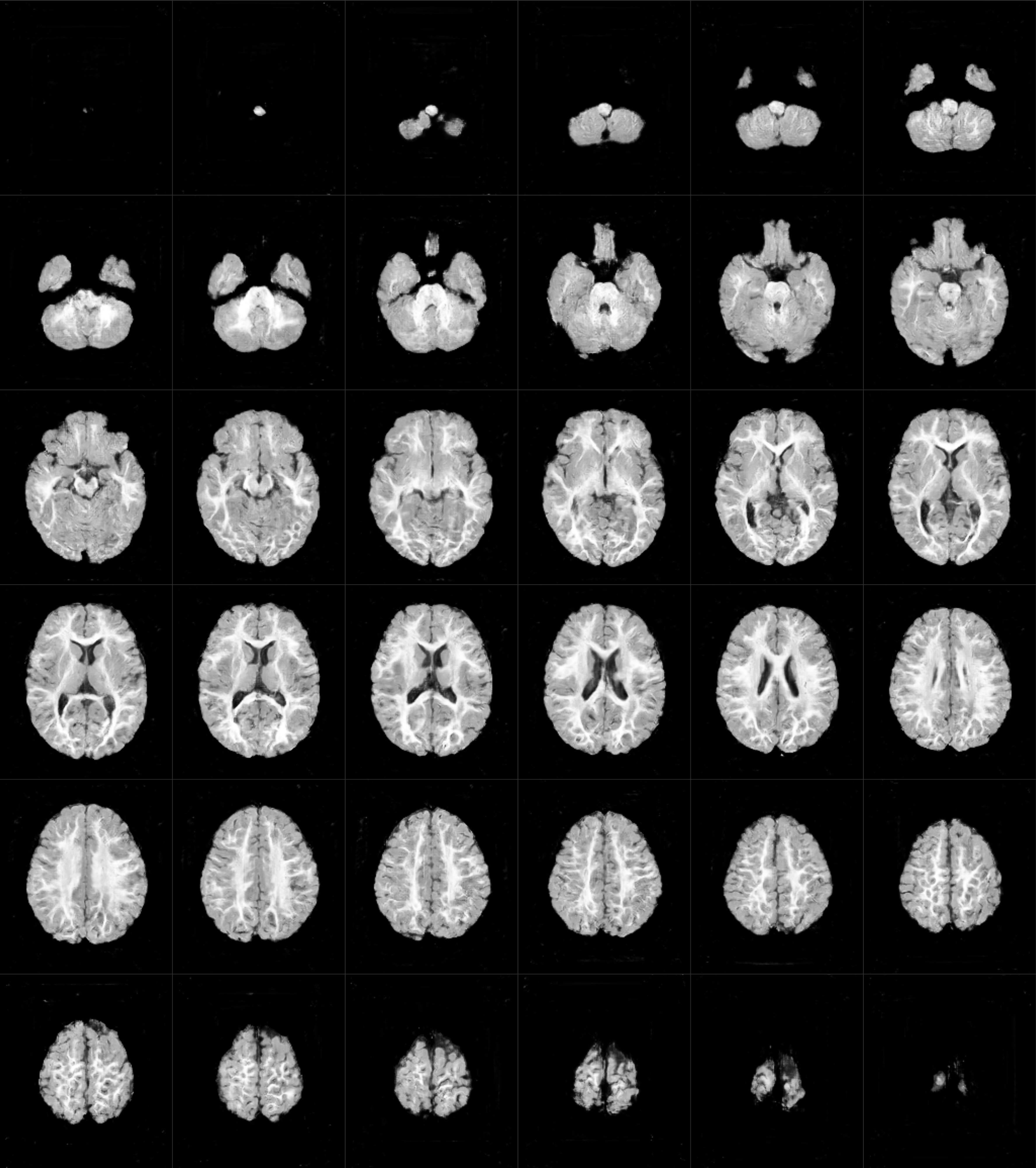

Supplement: Supplementary file 4 [file Data_Sheet_4.ZIP › 12monthFrom6MonthT1/12monthFrom6MonthT1_GAN.png]

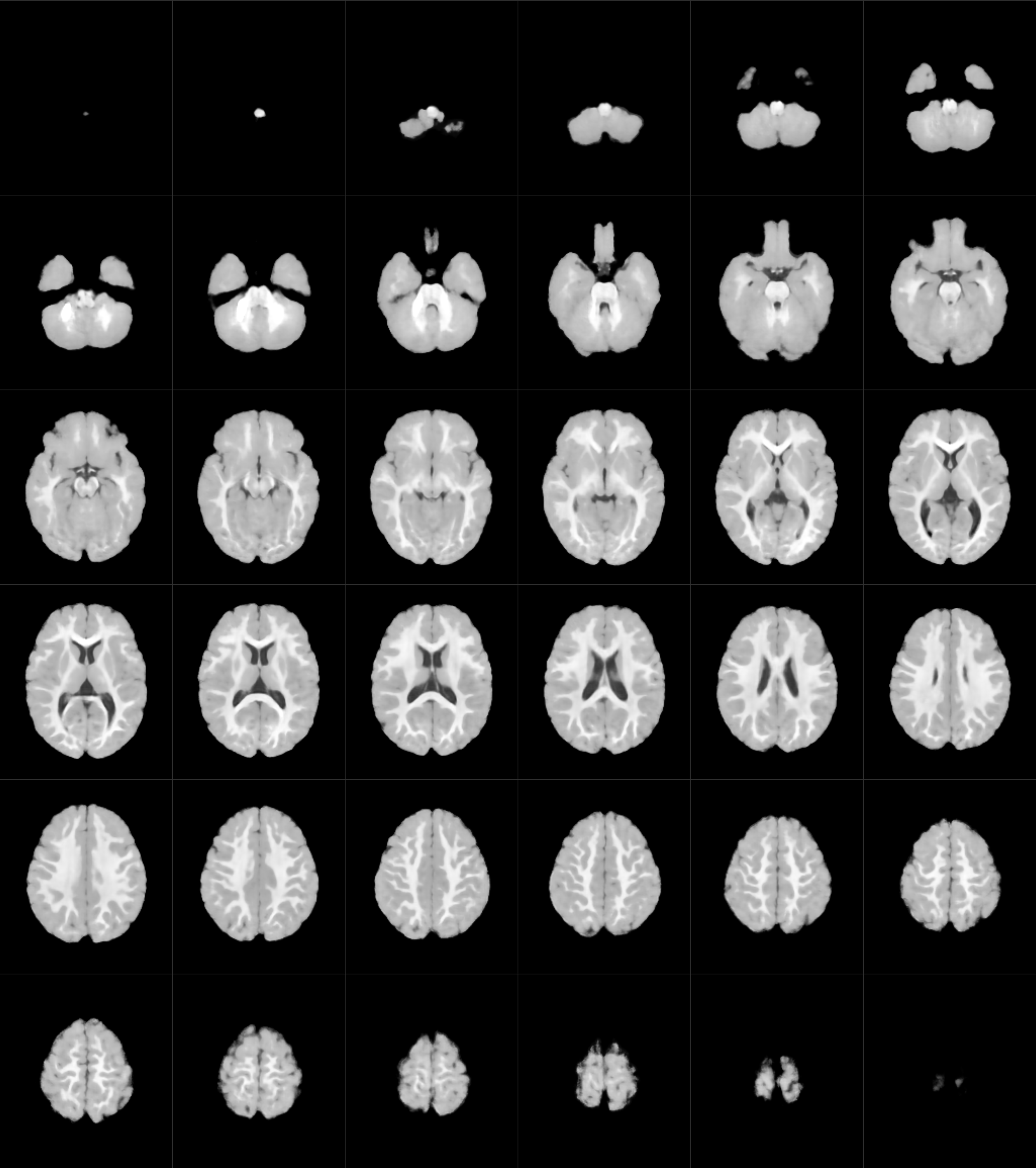

Supplement: Supplementary file 4 [file Data_Sheet_4.ZIP › 12monthFrom6MonthT1/12monthFrom6MonthT1_GAN_L1.png]

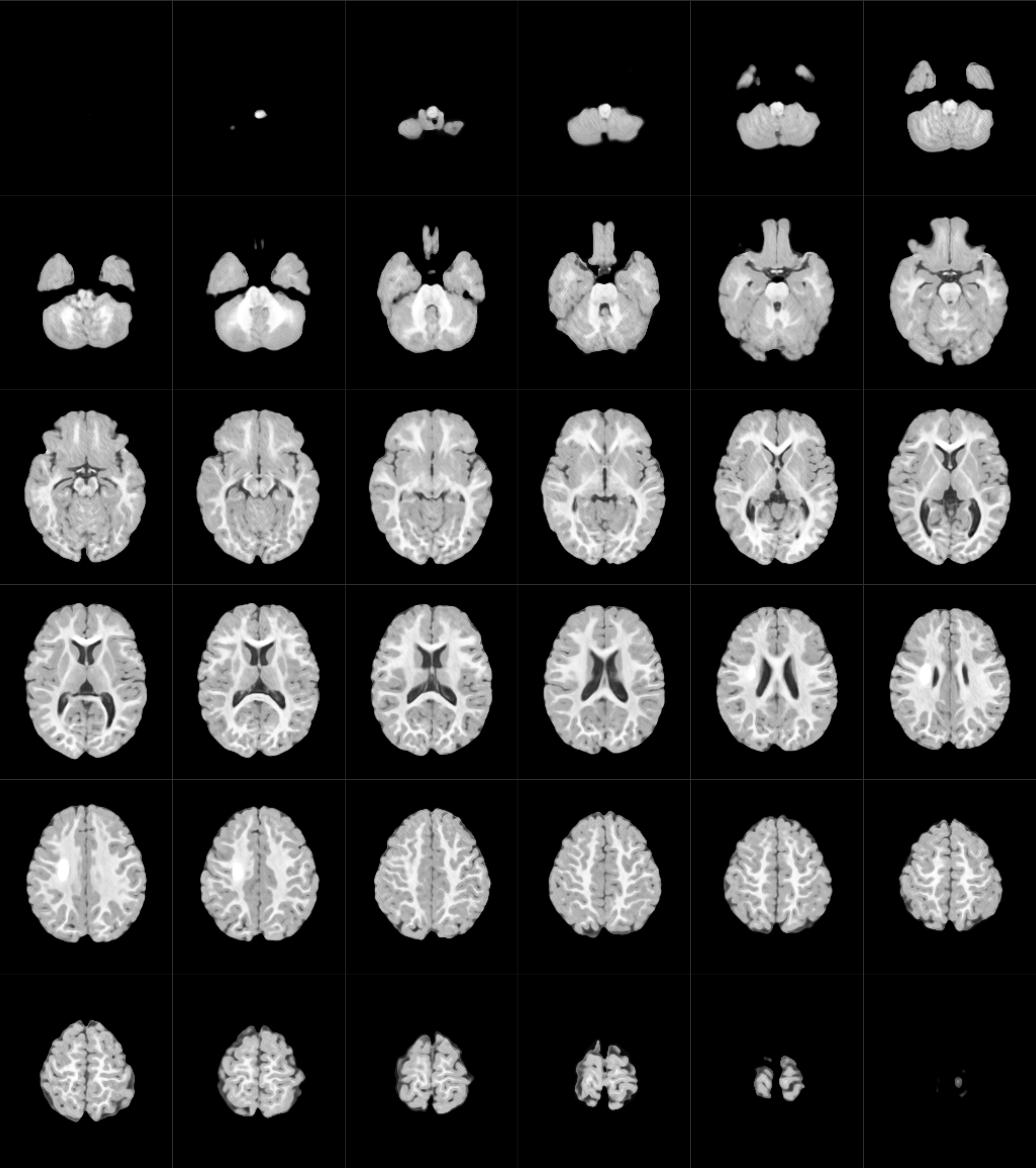

Supplement: Supplementary file 4 [file Data_Sheet_4.ZIP › 12monthFrom6MonthT1/12monthFrom6MonthT1_Unet_Lp.png]

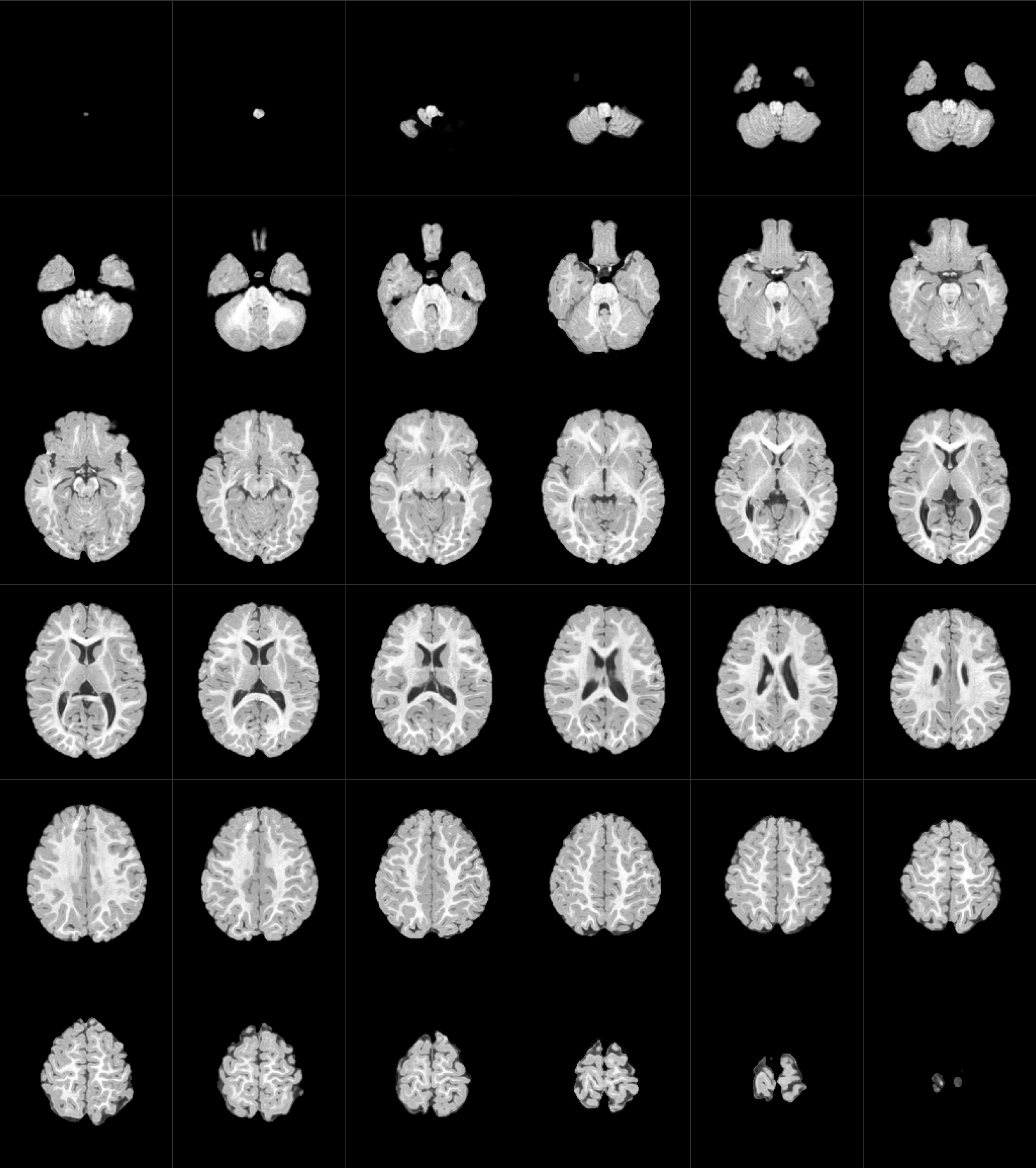

Supplement: Supplementary file 4 [file Data_Sheet_4.ZIP › 12monthFrom6MonthT1/12monthFrom6MonthT1_MPGAN.png]

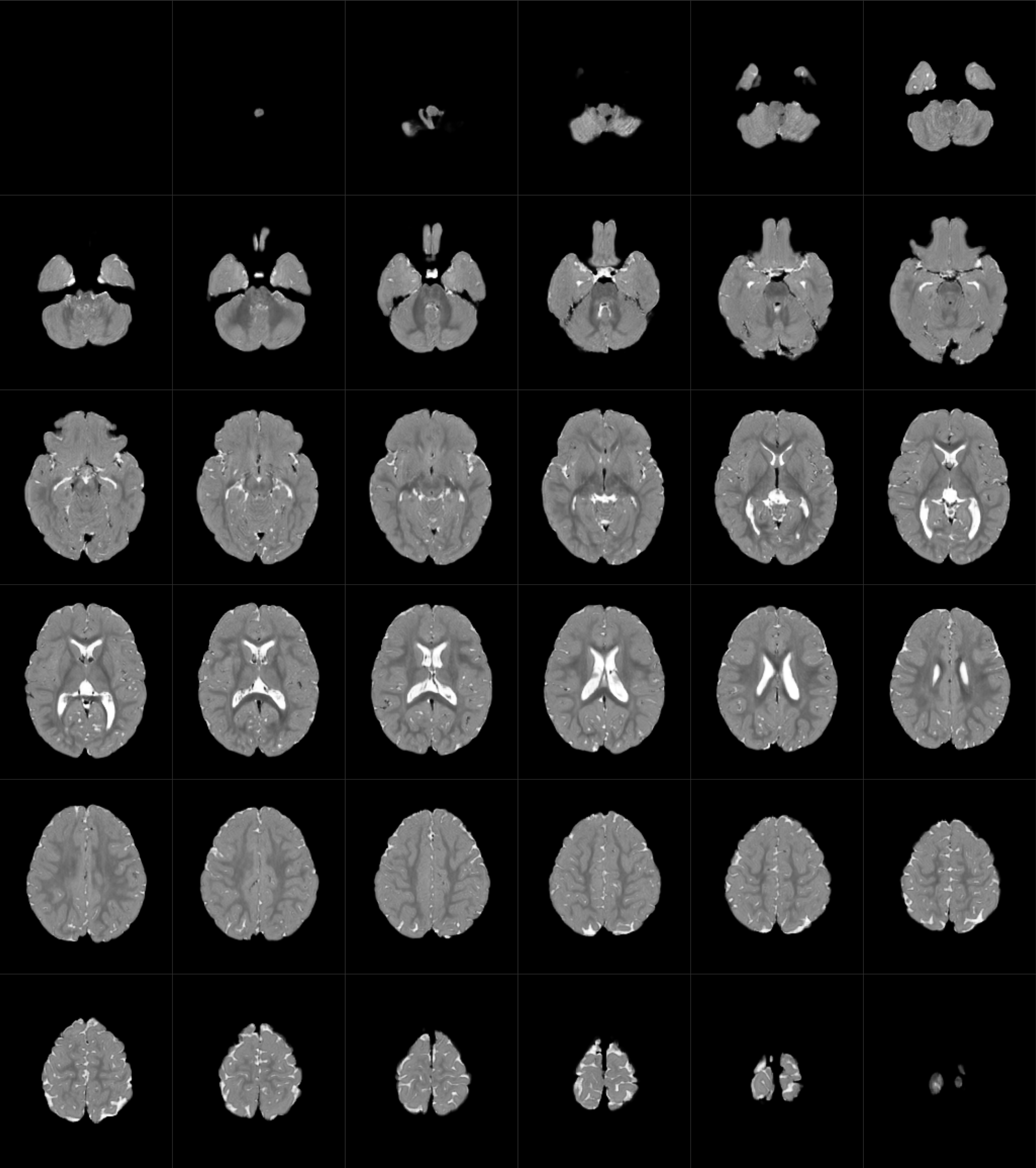

Supplement: Supplementary file 5 [file Data_Sheet_5.ZIP › 12monthFrom6MonthT2/12monthFrom6MonthT2_MPGAN.png]

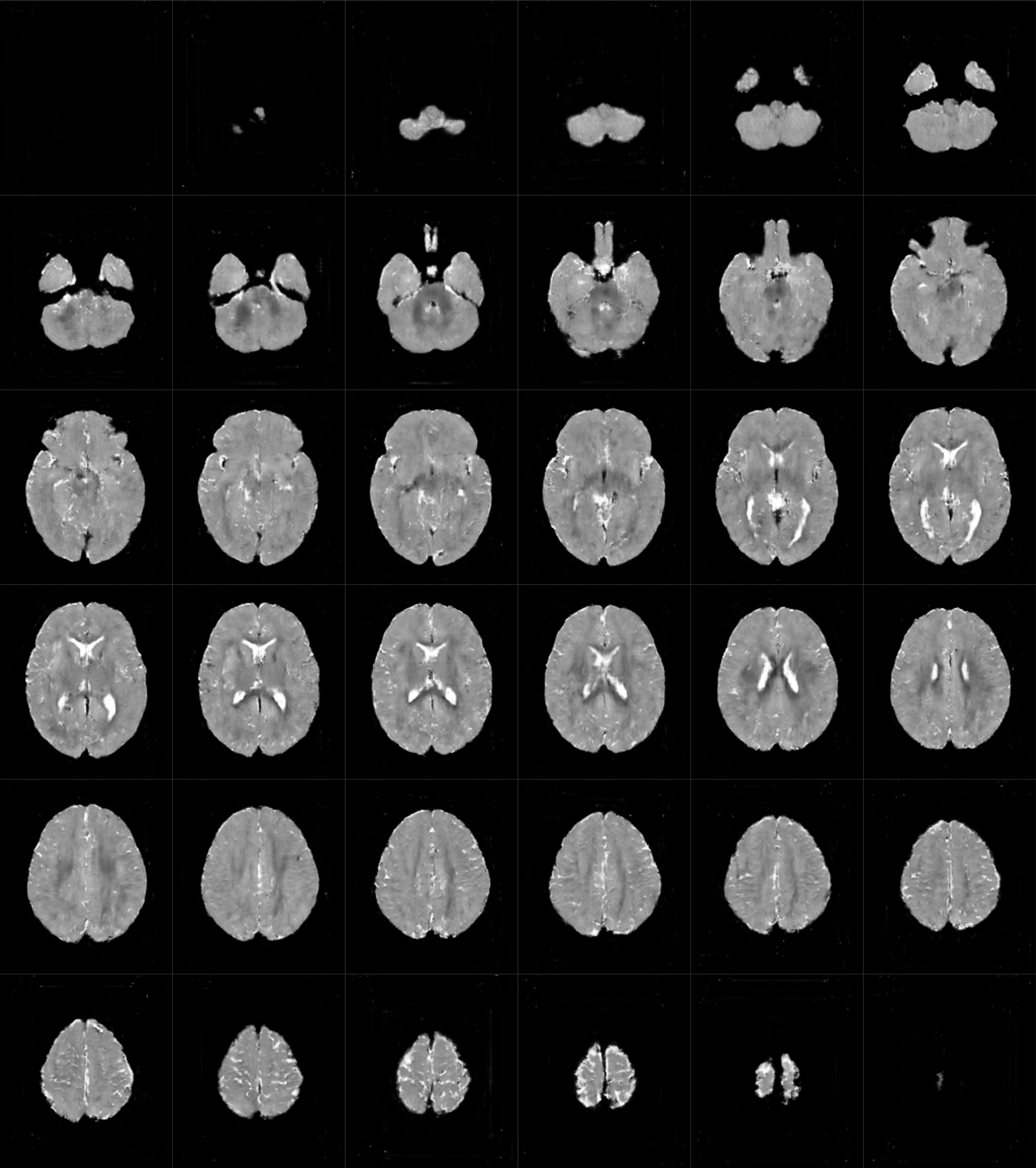

Supplement: Supplementary file 5 [file Data_Sheet_5.ZIP › 12monthFrom6MonthT2/12monthFrom6MonthT2_GAN.png]

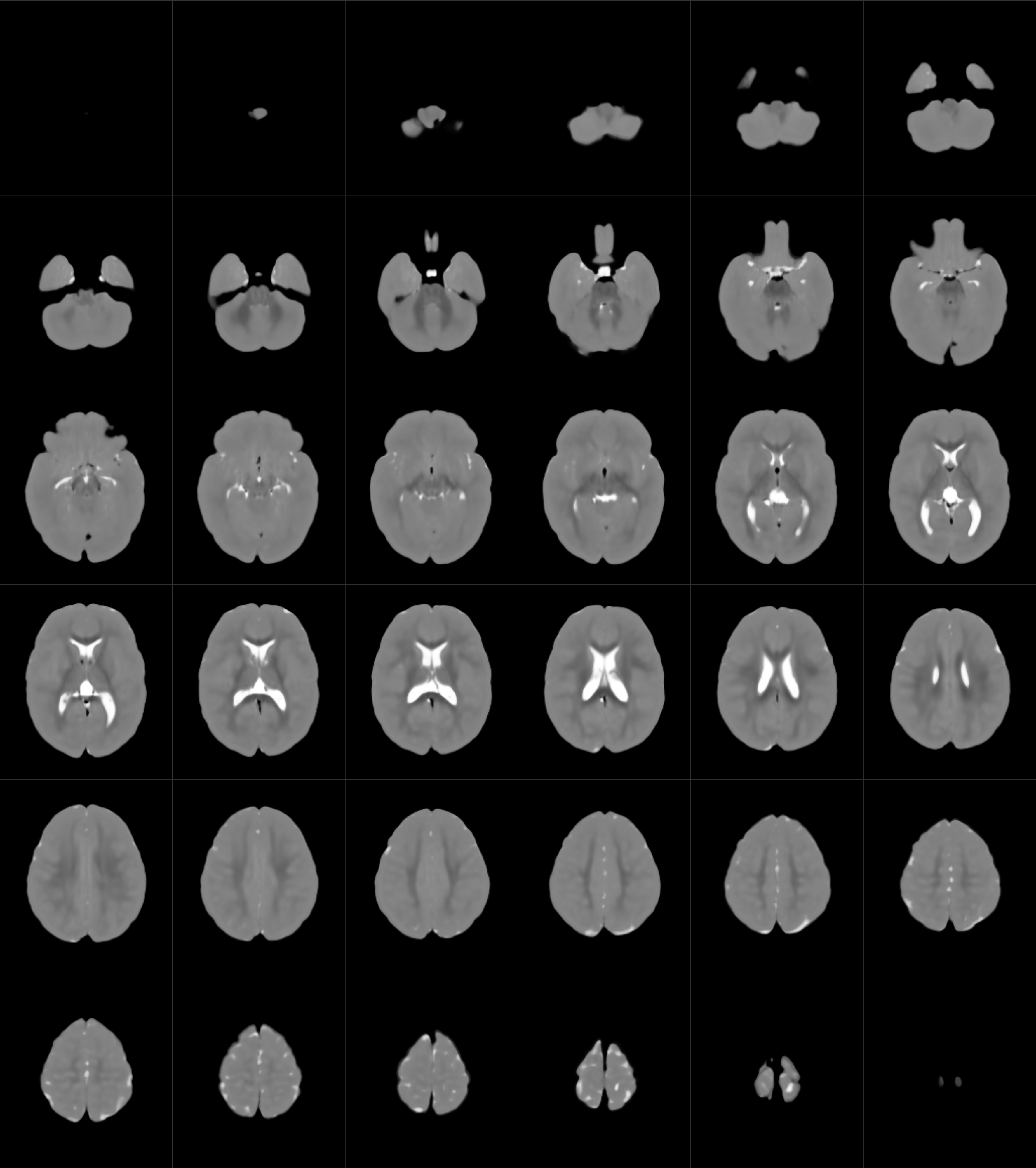

Supplement: Supplementary file 5 [file Data_Sheet_5.ZIP › 12monthFrom6MonthT2/12monthFrom6MonthT2_GAN_L1.png]

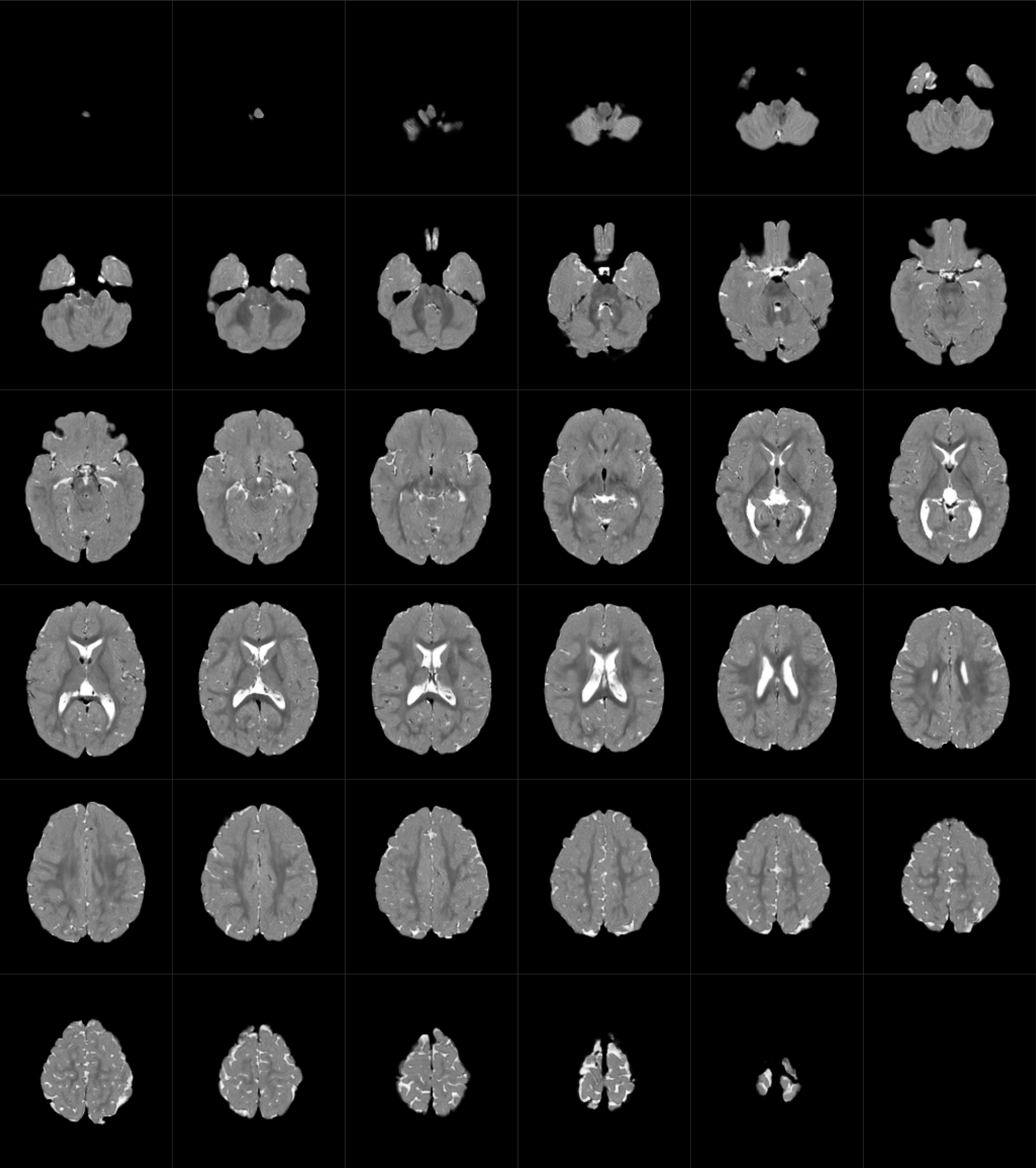

Supplement: Supplementary file 5 [file Data_Sheet_5.ZIP › 12monthFrom6MonthT2/12monthFrom6MonthT2_PGAN.png]

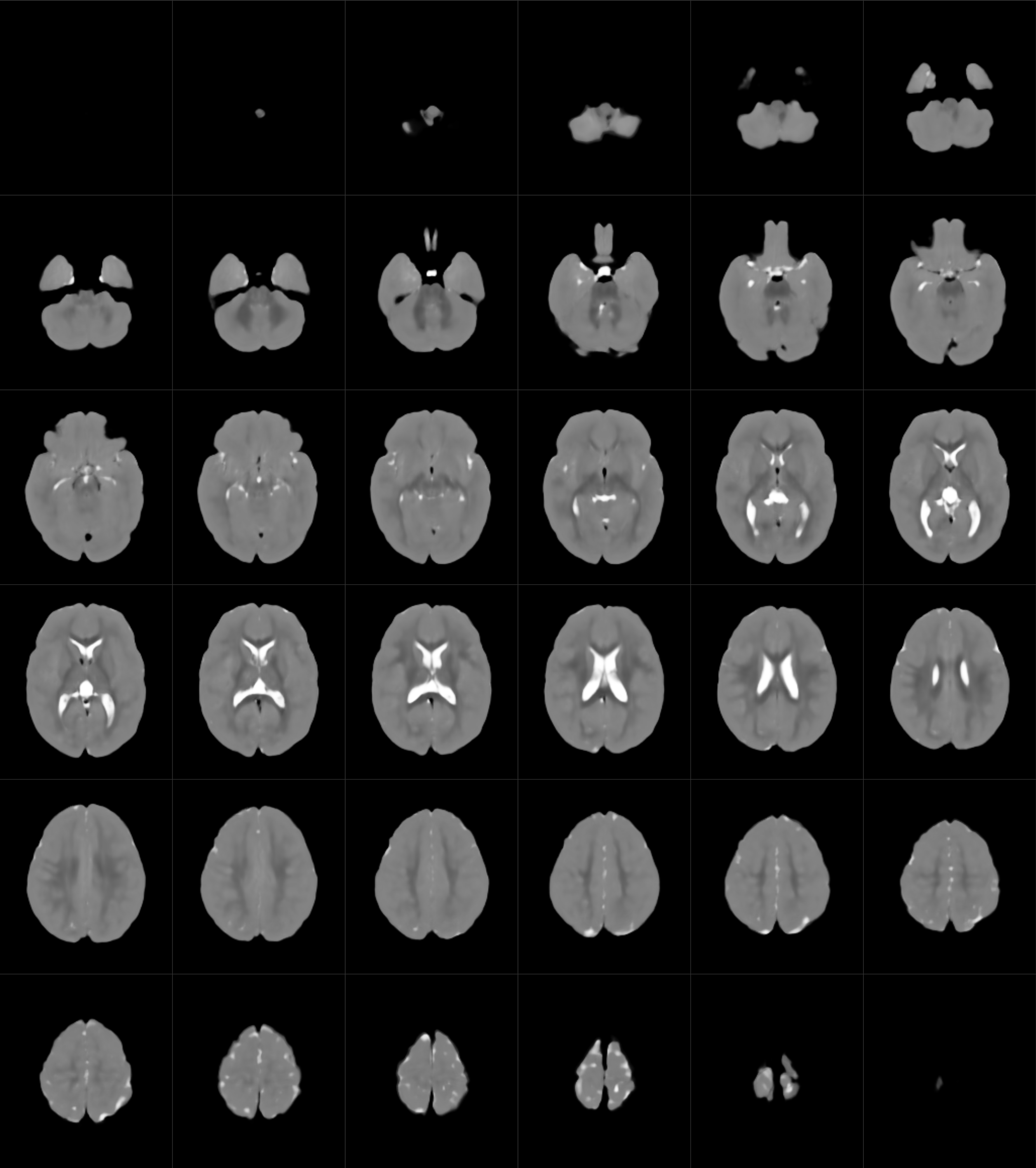

Supplement: Supplementary file 5 [file Data_Sheet_5.ZIP › 12monthFrom6MonthT2/12monthFrom6MonthT2_Unet.png]

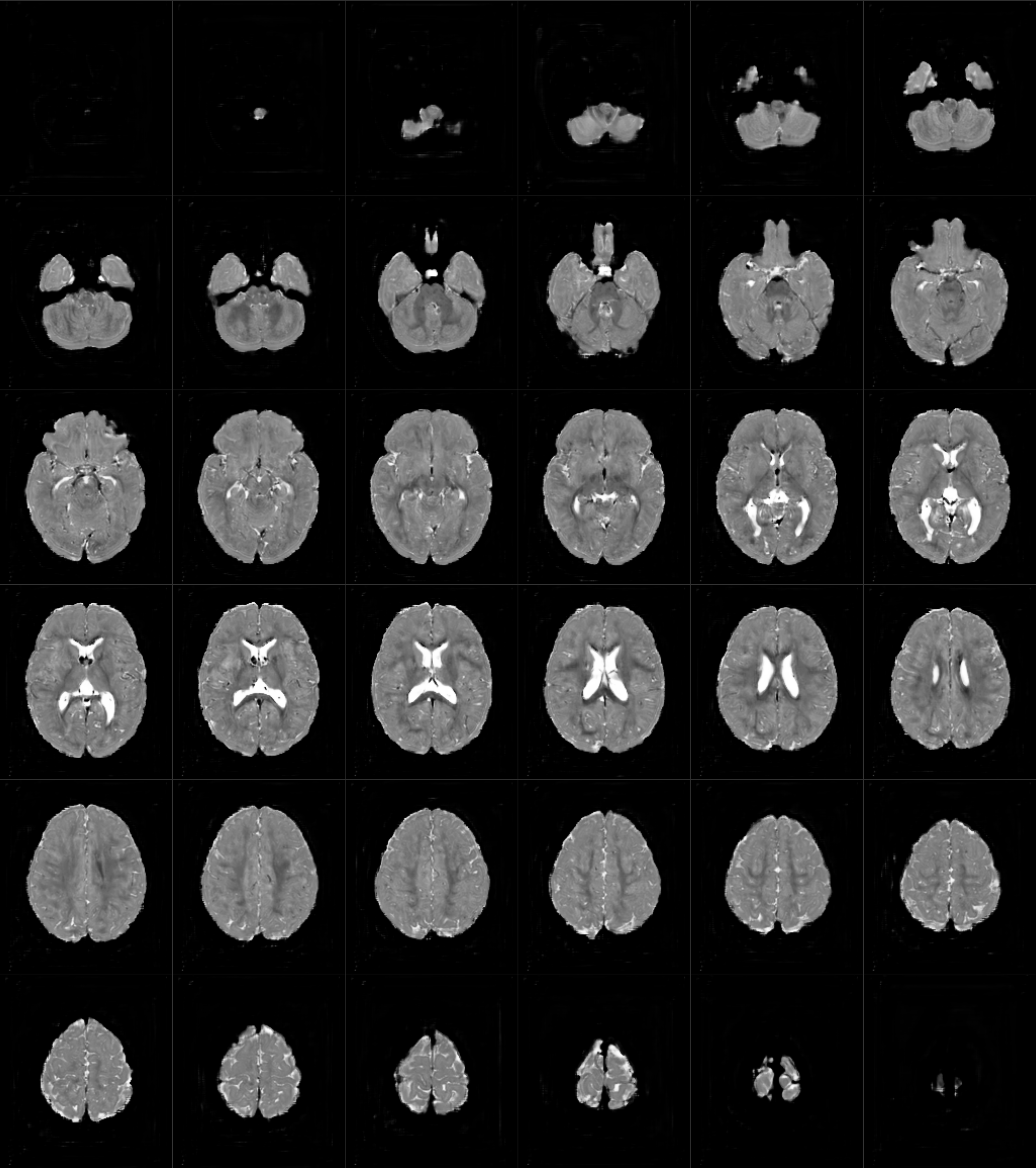

Supplement: Supplementary file 5 [file Data_Sheet_5.ZIP › 12monthFrom6MonthT2/12monthFrom6MonthT2_CycleGAN.png]

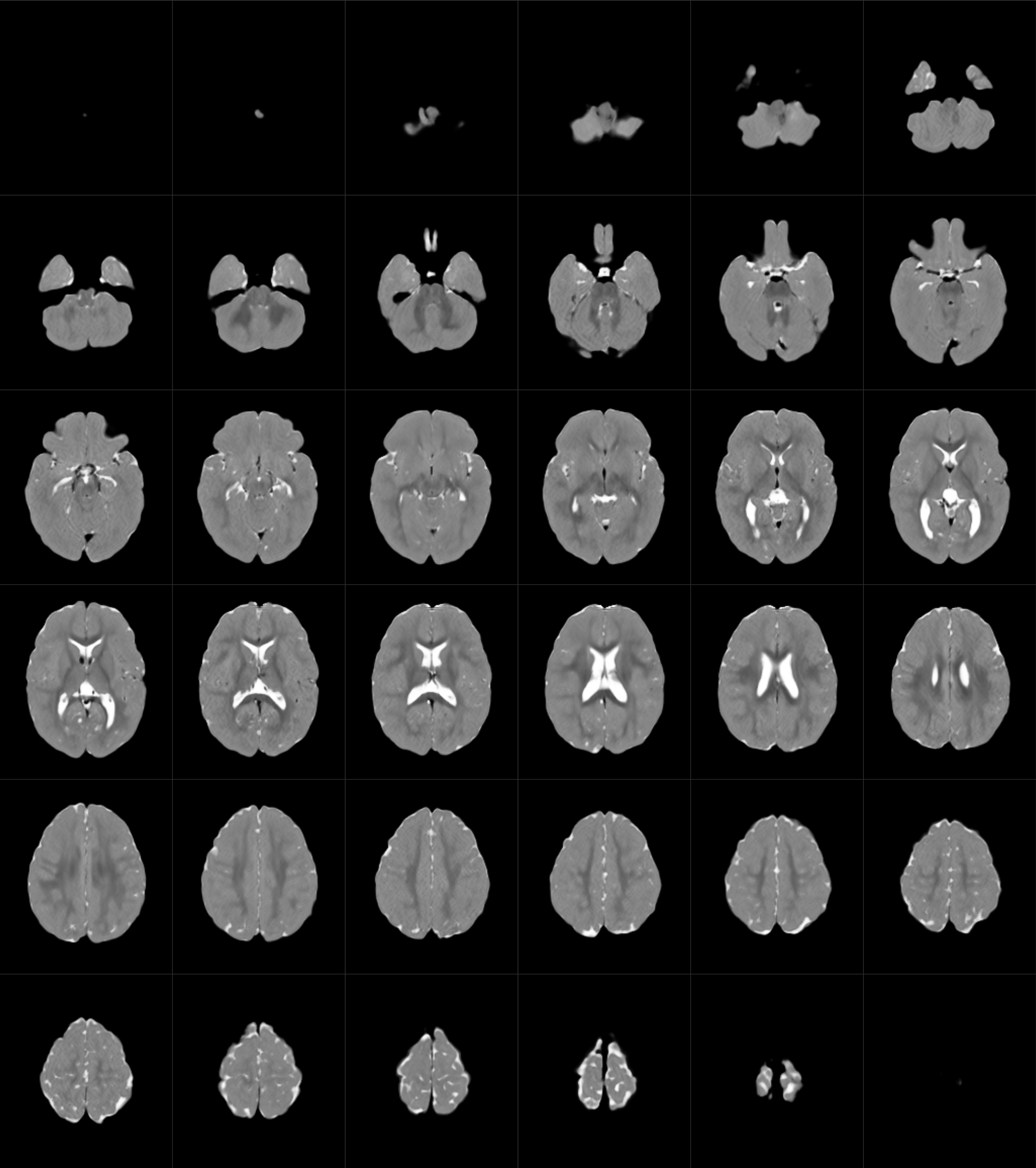

Supplement: Supplementary file 5 [file Data_Sheet_5.ZIP › 12monthFrom6MonthT2/12monthFrom6MonthT2_Unet_Lp.png]

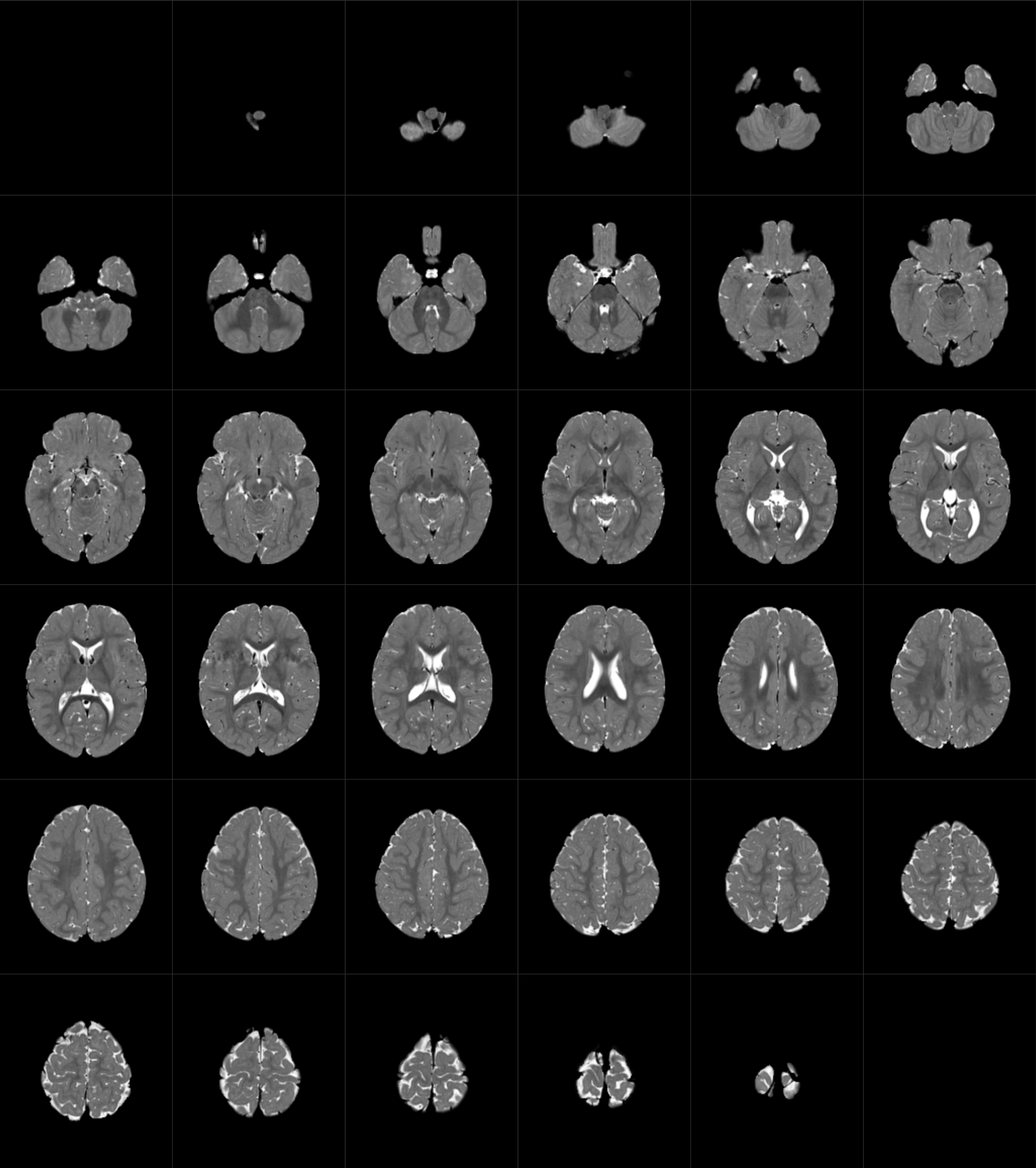

Supplement: Supplementary file 5 [file Data_Sheet_5.ZIP › 12monthFrom6MonthT2/12monthFrom6MonthT2_GroundTruth.png]
